# The unusual mode of action of the polyketide glycoside antibiotic cervimycin C

**DOI:** 10.1128/msphere.00764-23

**Published:** 2024-05-09

**Authors:** Alina Hoffmann, Ursula Steffens, Boris Maček, Mirita Franz-Wachtel, Kay Nieselt, Theresa Anisja Harbig, Kirstin Scherlach, Christian Hertweck, Hans-Georg Sahl, Gabriele Bierbaum

**Affiliations:** 1 University Hospital Bonn, Institute of Medical Microbiology, Immunology and Parasitology, Bonn, Germany; 2 University of Tübingen, Proteome Center Tübingen, Tübingen, Germany; 3 University of Tübingen, Interfaculty Institute for Bioinformatics and Medical Informatics, Tübingen, Germany; 4 Leibniz Institute for Natural Product Research and Infection Biology–Hans Knöll Institute (HKI), Jena, Germany; 5 Friedrich Schiller University Jena, Institute of Microbiology, Faculty of Biological Sciences, Jena, Germany; 6 University of Bonn, Institute for Pharmaceutical Microbiology, Bonn, Germany; University of Rochester, Rochester, New York, USA

**Keywords:** antibiotic, polyketide, gyrase, chromosome segregation, septum formation, mode of action, heat shock response, WalKR

## Abstract

Cervimycins A–D are bis-glycosylated polyketide antibiotics produced by *Streptomyces tendae* HKI 0179 with bactericidal activity against Gram-positive bacteria. In this study, cervimycin C (CmC) treatment caused a spaghetti-like phenotype in *Bacillus subtilis* 168, with elongated curved cells, which stayed joined after cell division, and exhibited a chromosome segregation defect, resulting in ghost cells without DNA. Electron microscopy of CmC-treated *Staphylococcus aureus* (3 × MIC) revealed swollen cells, misshapen septa, cell wall thickening, and a rough cell wall surface. Incorporation tests in *B. subtilis* indicated an effect on DNA biosynthesis at high cervimycin concentrations. Indeed, artificial downregulation of the DNA gyrase subunit B gene (*gyrB*) increased the activity of cervimycin in agar diffusion tests, and, in high concentrations (starting at 62.5 × MIC), the antibiotic inhibited *S. aureus* DNA gyrase supercoiling activity *in vitro*. To obtain a more global view on the mode of action of CmC, transcriptomics and proteomics of cervimycin treated versus untreated *S. aureus* cells were performed. Interestingly, 3 × MIC of cervimycin did not induce characteristic responses, which would indicate disturbance of the DNA gyrase activity *in vivo*. Instead, cervimycin induced the expression of the CtsR/HrcA heat shock operon and the expression of autolysins, exhibiting similarity to the ribosome-targeting antibiotic gentamicin. In summary, we identified the DNA gyrase as a target, but at low concentrations, electron microscopy and omics data revealed a more complex mode of action of cervimycin, which comprised induction of the heat shock response, indicating protein stress in the cell.

**IMPORTANCE:**

Antibiotic resistance of Gram-positive bacteria is an emerging problem in modern medicine, and new antibiotics with novel modes of action are urgently needed. Secondary metabolites from *Streptomyces* species are an important source of antibiotics, like the cervimycin complex produced by *Streptomyces tendae* HKI 0179. The phenotypic response of *Bacillus subtilis* and *Staphylococcus aureus* toward cervimycin C indicated a chromosome segregation and septum formation defect. This effect was at first attributed to an interaction between cervimycin C and the DNA gyrase. However, omics data of cervimycin treated versus untreated *S. aureus* cells indicated a different mode of action, because the stress response did not include the SOS response but resembled the response toward antibiotics that induce mistranslation or premature chain termination and cause protein stress. In summary, these results point toward a possibly novel mechanism that generates protein stress in the cells and subsequently leads to defects in cell and chromosome segregation.

## INTRODUCTION

The progressive spread of infections caused by antibiotic-resistant bacteria represents a serious health threat worldwide (1). Hence, new antibacterial agents, preferably with novel resistance-breaking modes of action, are urgently needed (2). Especially, methicillin-resistant *Staphylococcus aureus* (MRSA) strains and vancomycin-resistant enterococci (VRE) belong to a group of high-priority pathogens with urgent need for new antibiotics (2). In this regard, *Streptomyces* species are a promising origin of secondary metabolites including anti-MRSA drugs (3).

**Fig 1:**
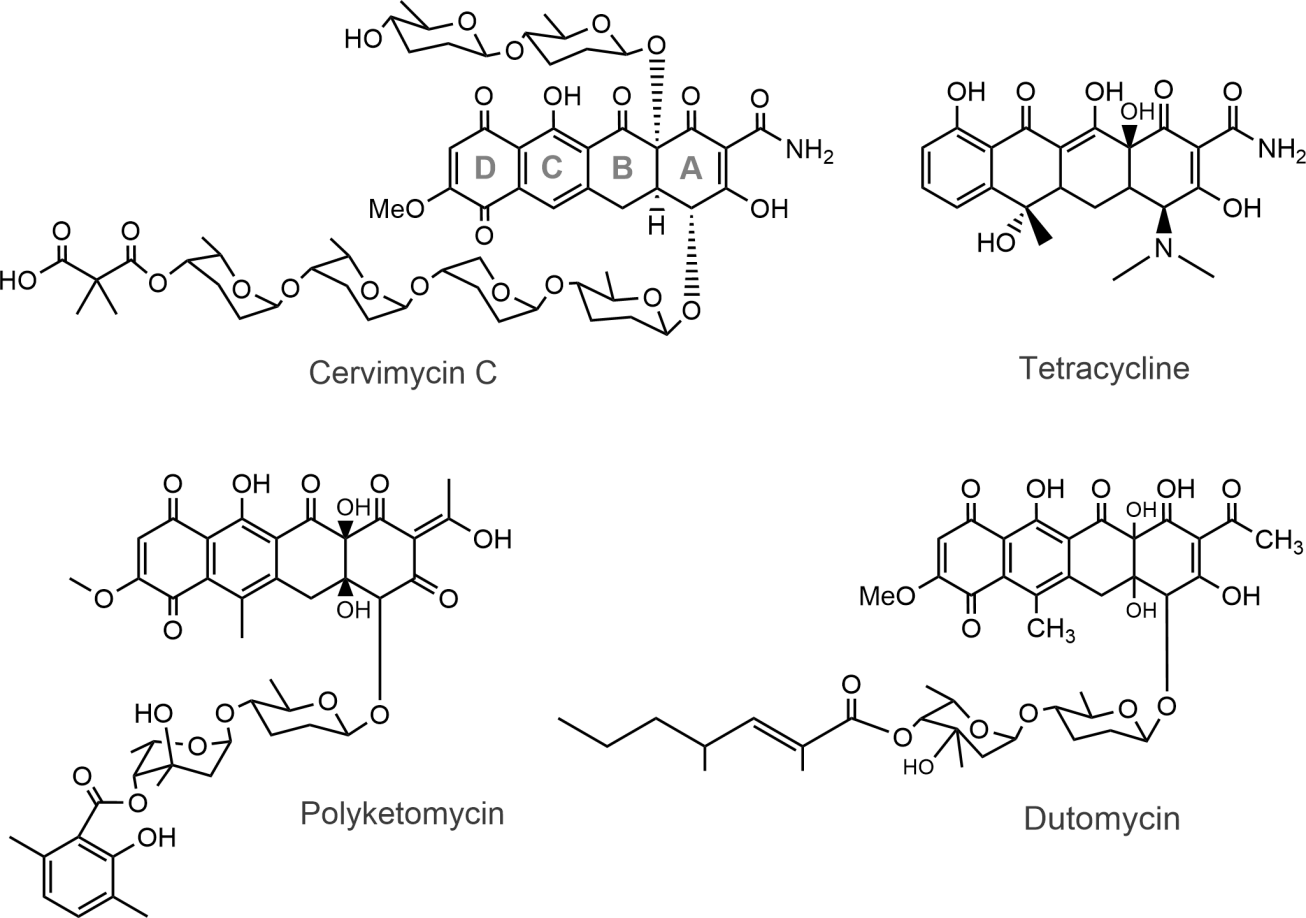
Structure of cervimycin C and comparison to related tetracyclic antibiotics (polyketomycin [5] and dutomycin [6]) and tetracycline.

The cervimycins are produced by *Streptomyces tendae* HKI 0179 and show potent activity against Gram-positive pathogens, including VRE and MRSA (4). The cervimycins possess a common tetracyclic polyketide core structure that is highly substituted with six sugar moieties (Fig. 1), and the components of the antibiotic complex (cervimycins A–M) differ in their substitution patterns (4). The core structure is reminiscent of the tetracyclines, and the anthracyclines polyketomycin (5) and dutomycin (6) are structurally related to cervimycin (Fig. 1). The spectrum of activity is limited to Gram-positive bacteria, and even *Escherichia coli* strains with an outer membrane defect remain resistant (Table 1, data from Dietrich et al. [7]).

**TABLE 1:** Minimal inhibitory concentrations (mg/L) of cervimycin C (CmC) and cervimycin D (CmD) against various bacteria

Strain	CmC	CmD
*B. subtilis* 168	0.25	0.5
*S. aureus* SG511 Berlin	2	32
*S. aureus* NCTC 8325-4	16	n.d.^*a*^
*E. coli* MB5746 (*lpxC*, *tolC*:Tn*10*)	>64	>64

^*a*^ n.d., not determined.

Although the polyphenolic structure could suggest DNA intercalation, this activity was not seen for cervimycins (8). Only very high cervimycin concentrations (50 µg/mL cervimycin A [8 × MIC]) inhibited the incorporation of radioactively labeled precursors into the DNA (8). In contrast, the biosyntheses of RNA, protein, and cell wall were not affected in incorporation experiments (8). In addition, there was no effect on reporter strains monitoring cell wall biosynthesis (using the *liaI* and *ypuA* promoters) and RNA biosynthesis (*helD*). Using the translation stalling reporter (*bmrC* promoter), an effect on protein biosynthesis was also not detected. A small upregulation was observed using the *yorB-*DNA biosynthesis reporter clone (7).

The selection for cervimycin C-resistant *Bacillus subtilis* yielded an efflux-based resistance mechanism, based on the overexpression of the BmrA ABC transporter (9). A selection for cervimycin C- and D-resistant *S. aureus* strains by serial subculturing with slowly increasing cervimycin concentrations for 15–28 d, followed by plating on agar with cervimycin (7), resulted in vancomycin-intermediately resistant strains (VISA) with loss of function mutations in the caseinolytic protease gene *clpP* or its cognate Clp ATPase gene *clpC*, and mutations in the essential histidine kinase gene *walK* (7). ClpP is the proteolytic core of the caseinolytic protease complex and possesses a key function in protein homeostasis, targeted degradation of proteins and of transcriptional regulators, generally maintaining protein quality and tightly controlling key regulatory proteins (10), thereby affecting a large number of processes. The Clp complex consists of the tetradecameric proteolytic chamber formed by ClpP and one of several hexameric ClpP ATPases (10). The cognate Clp ATPases, ClpX and ClpC in *S. aureus*, control the proteolytic activity of ClpP by translocating substrates into the proteolytic chamber (10). Mutations in ClpX have been associated with antibiotic resistance and turnover of the autolysin Sle1 (11–13). The functions of ClpC are less well defined; however, the protein is needed under thermal stress conditions and for the modulation of respiratory growth (14, 15). Whereas direct interactions of cervimycin C (CmC) with ClpP and ClpX could be excluded by *in vitro* experiments, this has not yet been shown for ClpC, but *B. subtilis* and *S. aureus* Δ*clpC* mutants did not show increased resistance to cervimycin (7). The WalK kinase is part of a two-component system and phosphorylates the response regulator WalR, which functions in the regulation of peptidoglycan maturation, cell wall turnover, cell separation, and protein secretion, as well as biofilm formation, and positively controls global autolytic activity, particularly via AtlA and the LytM endopeptidase (16). However, the alterations in *clpCP* and *walK* might exert compensatory effects, as a direct interaction with cervimycin was neither seen for ClpP nor for WalK, leaving the definitive target of cervimycin to be determined (7).

Here, we characterize cervimycin-treated cells, employing microscopy, transcriptomics, proteomics, and *in vitro* enzyme activity assays. In summary, the results point toward a possibly novel mechanism that generates protein stress within the cells and leads to defects in cell and chromosome segregation.

## RESULTS

### Cervimycin C treatment leads to ghost cell formation in *B. subtilis*

The bactericidal activity of cervimycin is restricted to Gram-positive bacteria (7), and time-kill curves confirmed that CmC kills *B. subtilis* 168 and *S. aureus* SG511 Berlin because colony-forming units (CFU) decreased upon cervimycin treatment, without recovery even after overnight incubation (Fig. S1).

**Fig 2:**
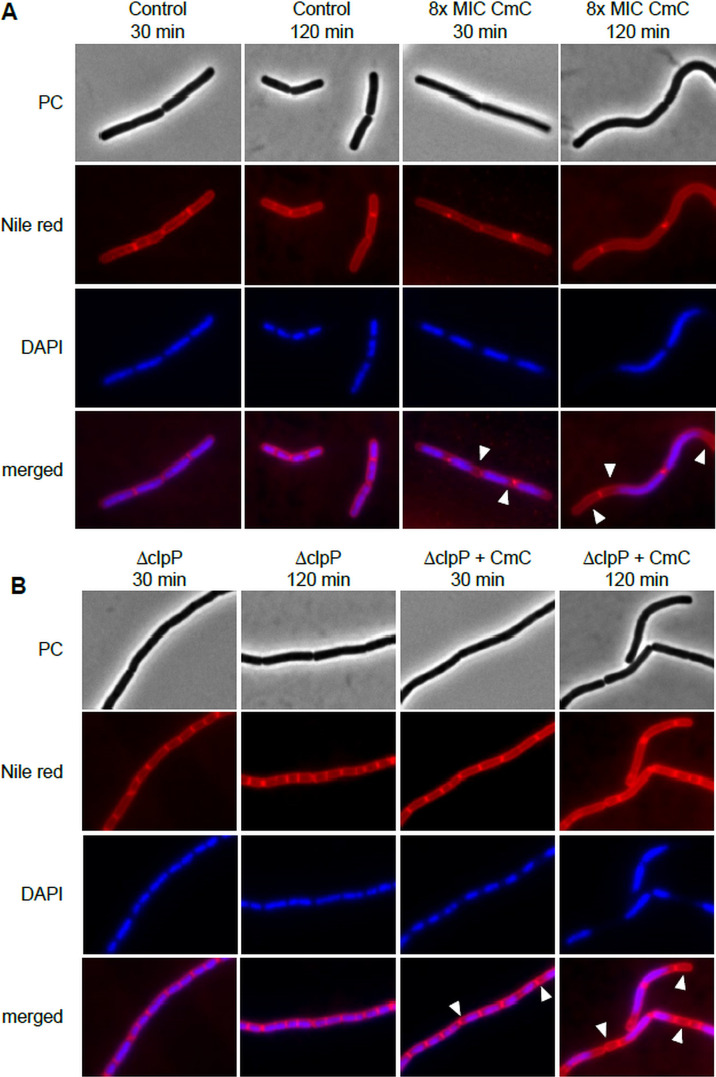
Fluorescence microscopy of cervimycin-treated *B. subtilis* 168 revealed a filamentation phenotype in combination with a chromosome segregation defect. (**A**) *B. subtilis* was grown in LB medium until early exponential growth phase and treated with 8 × MIC CmC or the same volume of DMSO (solvent control). At indicated time-points, DNA was visualized with DAPI, and the membrane was stained with Nile red. (**B**) A *B. subtilis clpP* deletion mutant exhibited the same chromosome segregation defect under CmC treatment as the wild type, but filamentation also occurred in the solvent control. PC, phase contrast; arrowheads, atypical partitioning of chromosome.

For further insight into the effect of CmC *in vitro*, *B. subtilis* 168 was grown in the presence and absence of 8 × MIC CmC, a concentration that had affected DNA biosynthesis in the incorporation experiments (8), and was visualized by fluorescence microscopy. Cells were stained with 4′,6-diamidino-2-phenylindole (DAPI) (DNA) and Nile red (membrane) (Fig. 2). Cervimycin treatment caused a spaghetti-like phenotype in *B. subtilis*, with elongated curved cells, which stayed joined after cell division. In addition, a chromosome condensation and a segregation defect occurred. The atypical partitioning of the chromosome caused “ghost cells” devoid of DNA, especially after prolonged incubation (Fig. 2, arrowheads).

Because characterization of cervimycin-resistant *S. aureus* strains (CmR strains) had revealed loss of ClpC or ClpP activity (7), a *Bacillus clpP* mutant was also tested. Here, chromosome partitioning was still disturbed (Fig. 2B), but the filamentation phenotype of the *clpP* mutant could not be attributed to cervimycin treatment, as filamentation was also observed in the *clpP* solvent control. This effect has been described before, and is thought to rely on accumulation of protein substrates of ClpP involved in cell morphology or cell division (17).

### Cervimycin treatment disturbs septum formation in *S. aureus*

**Fig 3:**
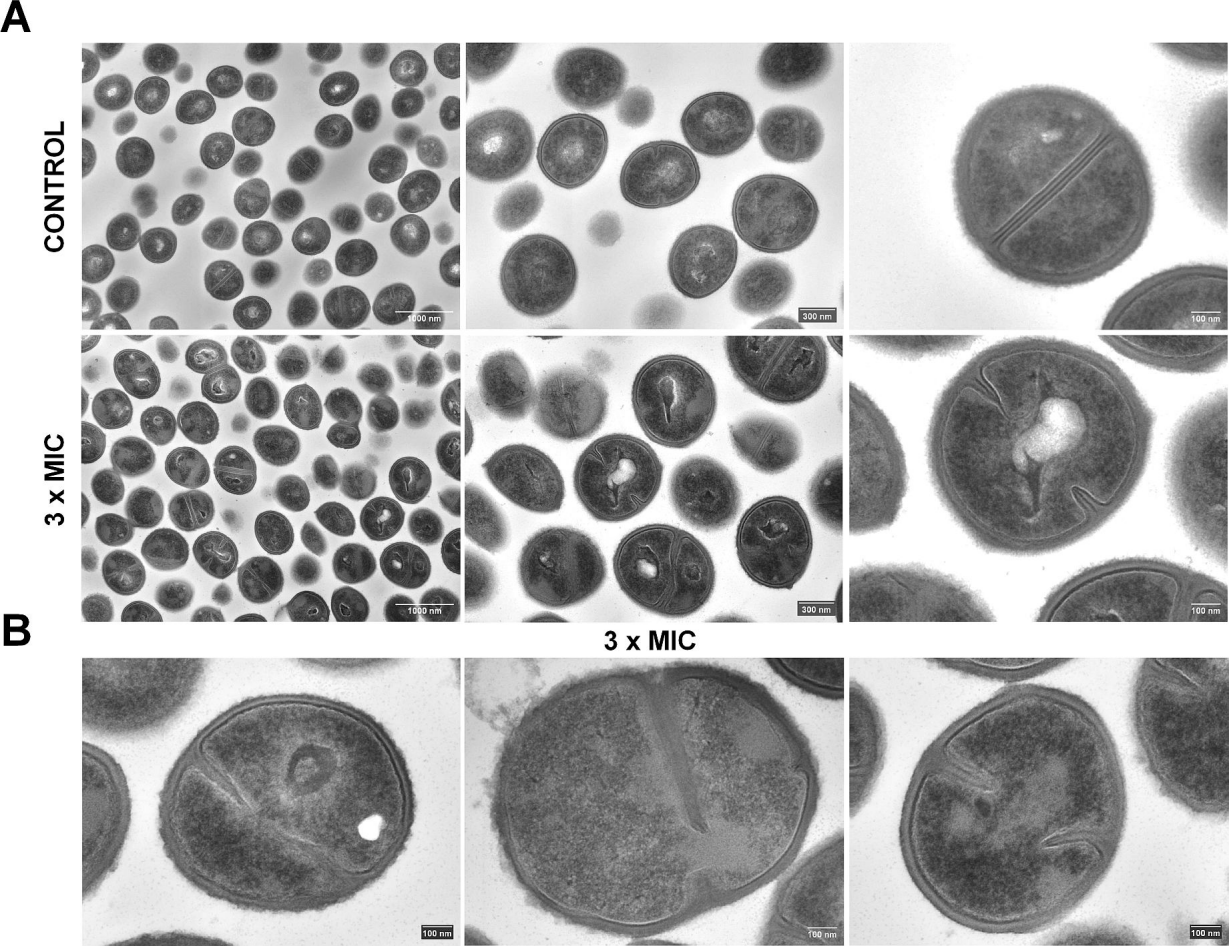
Scanning transmission electron microscopy (STEM) of *S. aureus* SG511 Berlin exposed to 3 × MIC CmC. (**A**) Comparison at different magnifications between unexposed (upper images) (7) and exposed samples to 3 × MIC CmC (lower images). Note the regularly rounded shape of the control cells and septa compared with the irregular morphology of the exposed cells. Scale bars: left, 1,000 nm; middle, 300 nm; right, 100 nm. (**B**) Close-ups of damaged cells exposed to CmC (3 × MIC). Note the misshapen septa, the thickening of the cell wall, and the rough cell wall surface. Scale bars: 100 nm.

Electron microscopy of cervimycin-treated *S. aureus* cells confirmed the cell division defect seen in *Bacillus* and revealed cell wall thickening after treatment with cervimycin (Fig. 3). Cervimycin treatment led to extensive defects in *S. aureus* cell morphology, causing irregularly shaped cells, including cell swelling, uneven separation of daughter cells, and sometimes a D-shape after daughter-cell separation was observed (Fig. 3). Second, the cell wall was significantly thickened (control: 34.91 ± 5.93 nm, 3 × MIC CmC: 46.06 ± 8.23 nm, Fig. S2) and the cell surface seemed rougher under cervimycin treatment, which indicates an imbalance between cell wall synthesis and cell wall hydrolysis. Third, various aspects of septum formation were disturbed under cervimycin treatment. Septa of irregular length, thickened septa, and thinning in the upper part of septa were visible. In some cases, the septa were bent, in contrast to the straight septa observed in the control. This effect may have been caused by the presence of the nucleoid, which was still located between the ingrowing septa, and the chromosome segregation effect observed in *Bacillus* supports this hypothesis.

### Influence of cervimycin on proteins involved in cell wall growth and chromosome segregation

Localization and activity of cell wall hydrolases in *S. aureus* are mediated by teichoic acids (18, 19). Inhibition of teichoic acid biosynthesis would explain the restriction of cervimycin antibacterial activity to Gram-positive bacteria. In this case, the deletion of *tagO*, the initiator of wall teichoic acid (WTA) biosynthesis (20), would render *S. aureus* cervimycin resistant. However, the deletion of *tagO* had only a minor effect on the cervimycin MIC (Table 2).

**TABLE 2:** Susceptibility of *tagO* and *recU* deletion strains to cervimycin^*a*^

Strain	Description	MIC (µg/mL)
SA113	Wild type	6
SA113 Δ*tagO*	Glycosyl transferase; initial step in WTA biosynthesis	3
NCTC 8325-4	Wild type	4–8
NCTC 8325-4*∆recU*	Holliday junction-specific endonuclease	4

^*a*^ Susceptibility of both deletion strains was determined via broth dilution in Müller-Hinton medium after 24 h of incubation.

The phenotype of cervimycin-treated cells was reminiscent of the growth defect seen in *recU*-depleted *S. aureus* cells (21). RecU is a Holliday junction resolvase, encoded in the same operon as penicillin-binding protein 2, and is required for correct chromosome segregation and DNA damage repair, and its absence leads to the formation of cells with septa bisecting the DNA, compact nucleoids, and anucleate cells (21). In spite of the similar phenotypic defects caused by cervimycin or by *recU* deletion, the *recU* deletion did not affect cervimycin susceptibility (Table 2).

### High concentrations of cervimycin inhibit *E. coli* and *S. aureus* DNA gyrase

**Fig 4:**
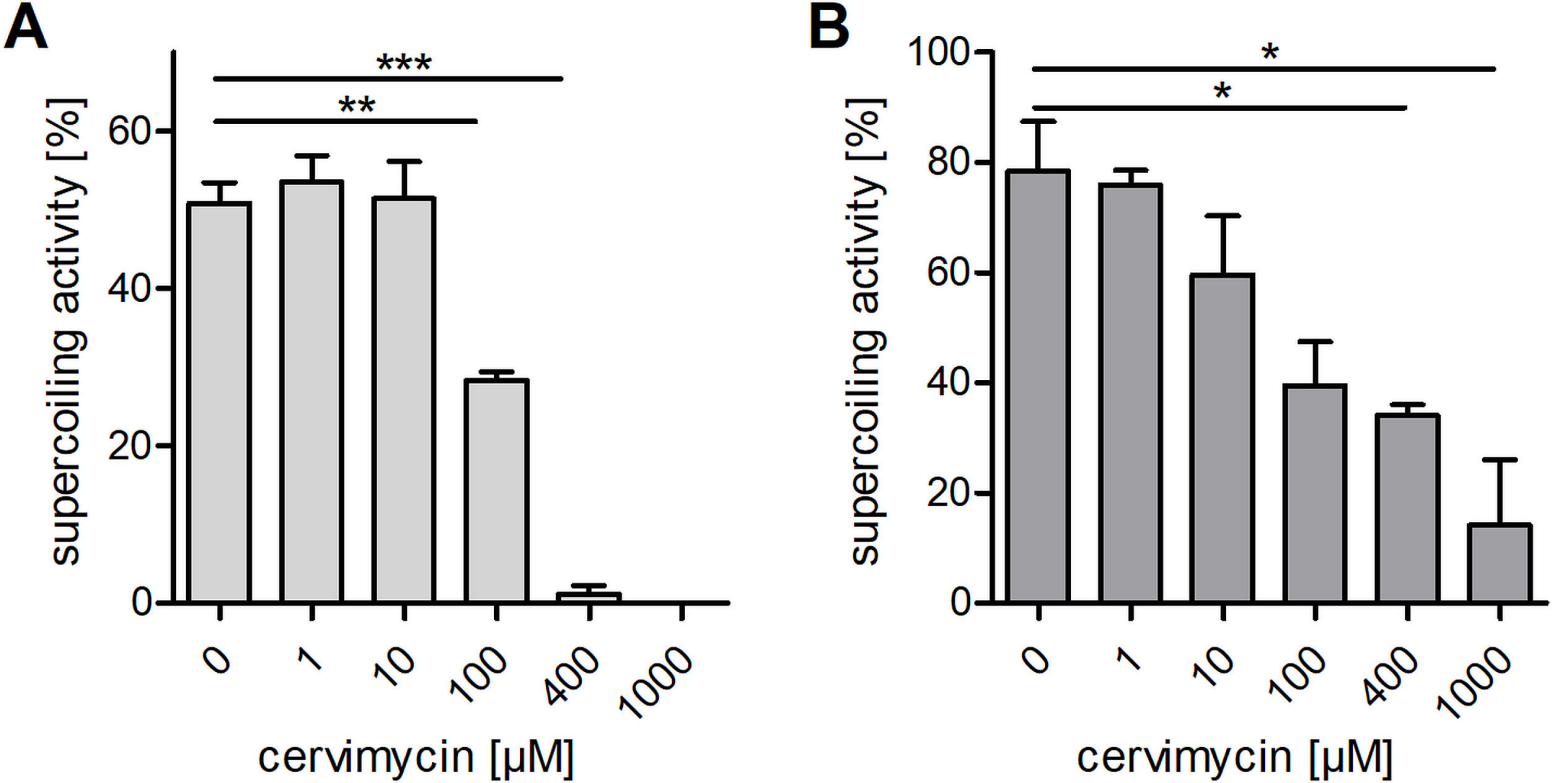
CmC inhibits *S. aureus* and *E. coli* DNA gyrase supercoiling activity in a concentration-dependent manner. (**A**) *S. aureus* and (**B**) *E. coli* DNA gyrase supercoiling activity were tested with increasing concentrations of cervimycin (*, *P* ≤ 0.05; **, *P* ≤ 0.0014; ***, *P <* 0.0001). Exemplary results of the gel-based activity assays are shown in Figure S4. At least 100-µM cervimycin was necessary to significantly decrease the DNA gyrase activity, which corresponds to 125 µg/mL CmC or the 62.5 × MIC of *S. aureus* SG511 Berlin.

Previous incorporation tests with radioactively labeled precursors (8) and a weak response of *B. subtilis yorB* (22) firefly luciferase reporter strain (7) had indicated that cervimycin might interfere with the DNA metabolism. Considering this and the inhibitory effect of high cervimycin concentrations on the incorporation of radioactive-labeled precursors into the DNA (8), DNA topoisomerases might constitute a possible target of cervimycin. DNA topoisomerases are essential enzymes, which alter the topology of the DNA during the cell cycle. Topoisomerases can redundantly relax supercoiled DNA, but DNA gyrase is unique and essential in introducing negative supercoils into the DNA in an ATP-dependent manner (23, 24). In order to gain a first insight into the effect of cervimycin on gyrase/topoisomerase activity, the susceptibility of clones expressing antisense RNA to *gyrA*, *gyrB*, *parC*, and *parE* to CmC was screened. Indeed, and in contrast to other topoisomerase genes, downregulation of the B subunit of the DNA gyrase (*gyrB*) increased susceptibility toward cervimycin (Fig. S3; Table 3). DNA gyrase inhibitors either bind the A subunit of the enzyme complex, like the fluoroquinolones ciprofloxacin and norfloxacin, leading to double-strand breaks and the induction of the SOS response (25, 26), or bind the B subunit, like novobiocin, leading to relaxation of the DNA and increased expression of the *recF-gyrA-gyrB*, *rib*, and *ure* operons (27–30). In order to assess the effect of CmC on gyrase activity, we next tested the DNA gyrase supercoiling activity in the absence and presence of CmC *in vitro* (Fig. 4; Fig. S4). Interestingly, both *S. aureus* and *E. coli* DNA gyrase were inhibited by high cervimycin concentrations, but *S. aureus* DNA gyrase was more susceptible to inhibition by cervimycin (Fig. 4). However, norfloxacin completely abolished *S. aureus* DNA gyrase supercoiling activity at a concentration of 10 µM (corresponding to 6.34 µg/mL or the 25 × MIC of *S. aureus* SG511 Berlin) (Fig. S4); in contrast, 400-µM cervimycin was needed to achieve this effect (corresponding to 500 µg/mL CmC or the 250 × MIC of *S. aureus* SG511 Berlin). Mutations in the DNA gyrase genes *gyrA* and *gyrB* were not detected during the selection of cervimycin-resistant *S. aureus* mutants (7), which contradicts the hypothesis of the DNA gyrase as primary target of cervimycin. We tested two *S*. *aureus* strains, which were resistant toward norfloxacin or novobiocin due to amino acid exchanges in the DNA gyrase. However, these strains did not exhibit cross-resistance toward cervimycin (Table S1), indicating different binding sites of these antibiotics on the DNA gyrase.

**TABLE 3:** GyrB- but not GyrA-depletion sensitizes *S. aureus* to cervimycin^*a*^

Gene	Function	Xylose IC_20_ (mM)	Fold increase of diameter of inhibition zone
*gyrA*	DNA gyrase subunit A	6.6	1.15
*gyrB*	DNA gyrase subunit B	17	1.45
*parC*	DNA topoisomerase IV subunit A	7.5	1.15
*parE*	DNA topoisomerase IV subunit B	17	1.15
*topA*	DNA topoisomerase I	6.6	1.12
*topB*	DNA topoisomerase III	10.5	1.00
*divIVA*	Cell division initiation protein DivIVA	10.5	0.86

^*a*^ Expression levels of tested genes were decreased by synthesis of antisense RNA using the pEPSA5 system using a xylose concentration leading to a 20% inhibition of expression (IC_20_) of the target gene (31). Cervimycin susceptibility was tested in agar diffusion assays on LB agar in comparison to an empty vector control.

Interestingly, GyrB shares the ATP-binding motif with another essential protein, namely, the WalK kinase, and cervimycin-resistant *S. aureus* mutants carried single-nucleotide polymorphisms (SNPs) in the essential *walK* gene. The WalRK two-component system, which comprises WalK and its cognate response regulator WalR, positively controls global autolytic activity (16), and mutations in the *wal* locus are often observed in mutants resistant toward the cell envelope targeting compounds vancomycin or daptomycin (32–38). Although a direct inhibition of WalK by cervimycin could not be demonstrated in earlier experiments (7), the cell wall thickening (Fig. S2) and the rough cell wall surface under cervimycin treatment (Fig. 3) might indicate that cervimycin interferes with cell wall turnover.

### Omics analyses reveal an extensive response of *S. aureus* toward cervimycin

**Fig 5:**
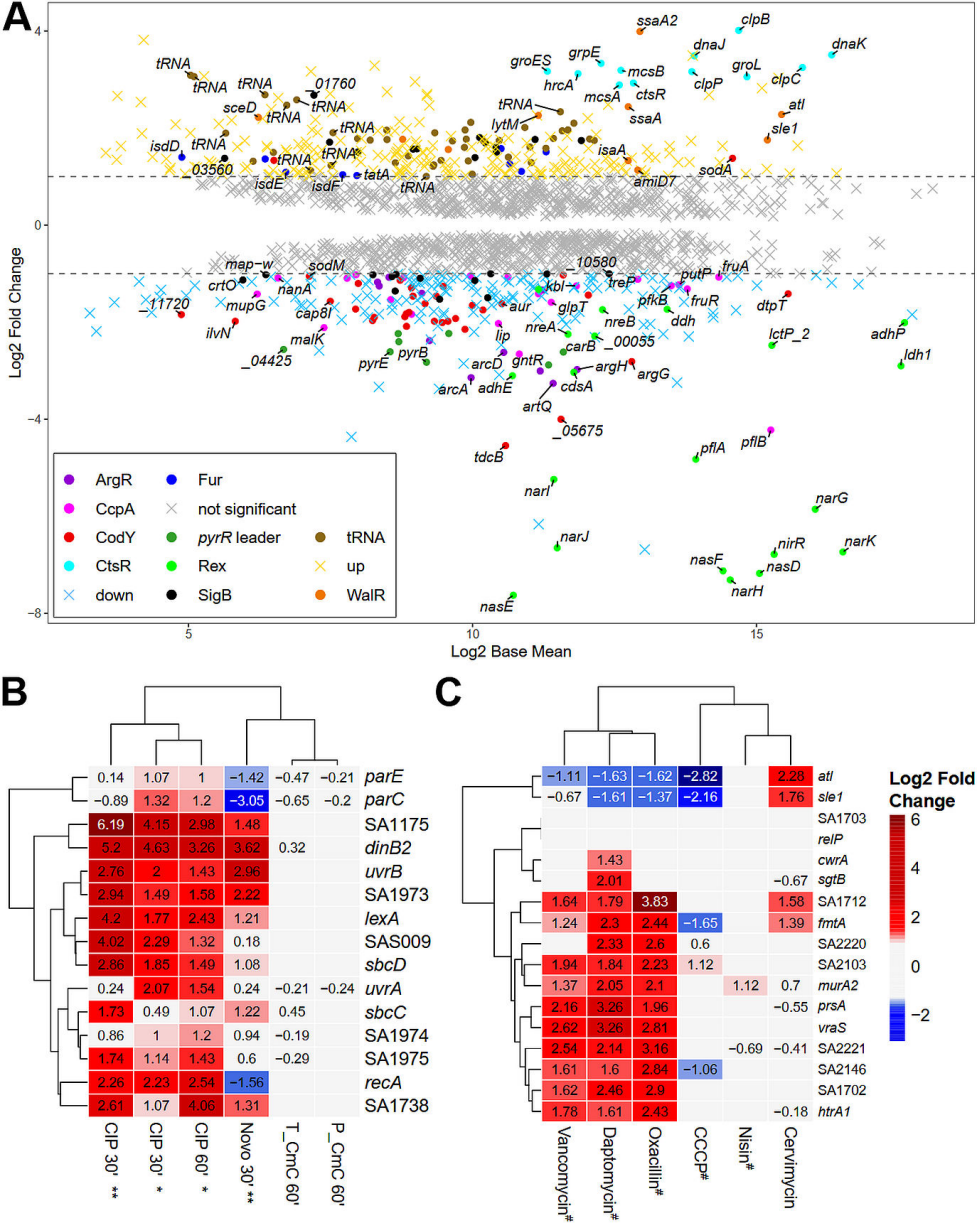
RNA-seq transcriptomics of *S. aureus* SG511 treated with 3 × MIC CmC for 60 min. (**A**) M/A plot. The expression profile is shown as ratio/intensity scatter plot (M/A plot: M value log_2_ FC, A value log_2_ base mean), which is based on the differential gene expression analysis. Colored symbols indicate significantly induced or repressed transcripts (M value ≥ 1 or ≤ −1; *P* value ≤ 0.05). Regulons with at least eight differentially expressed genes and tRNA genes are color coded. Colors refer to the annotated regulator (based on the AureoWiki Database [39]). Light gray symbols denote transcripts with similar expression levels in comparison to the untreated *S. aureus* SG511 Berlin (*P* value > 0.05, log_2_ FC ≤ 1 or ≥ −1). The RNA-seq data of differential transcription of all genes and regulons of the cervimycin-treated cells are listed in Table S3. Expression profiles of the SOS response genes (**B**), as induced by ciprofloxacin (*data from Cirz et al. [26]; **data from Jones et al. [40]), and the cell wall stress response genes (**C**), as induced by different cell wall and membrane active compounds (^#^data from McAleese et al. [41]), are compared to the response to CmC. Upregulation (log_2_ FC ≥ 1, red color) and downregulation (log_2_ FC ≤ −1, blue color) of the transcriptomic level are shown.

**Fig 6:**
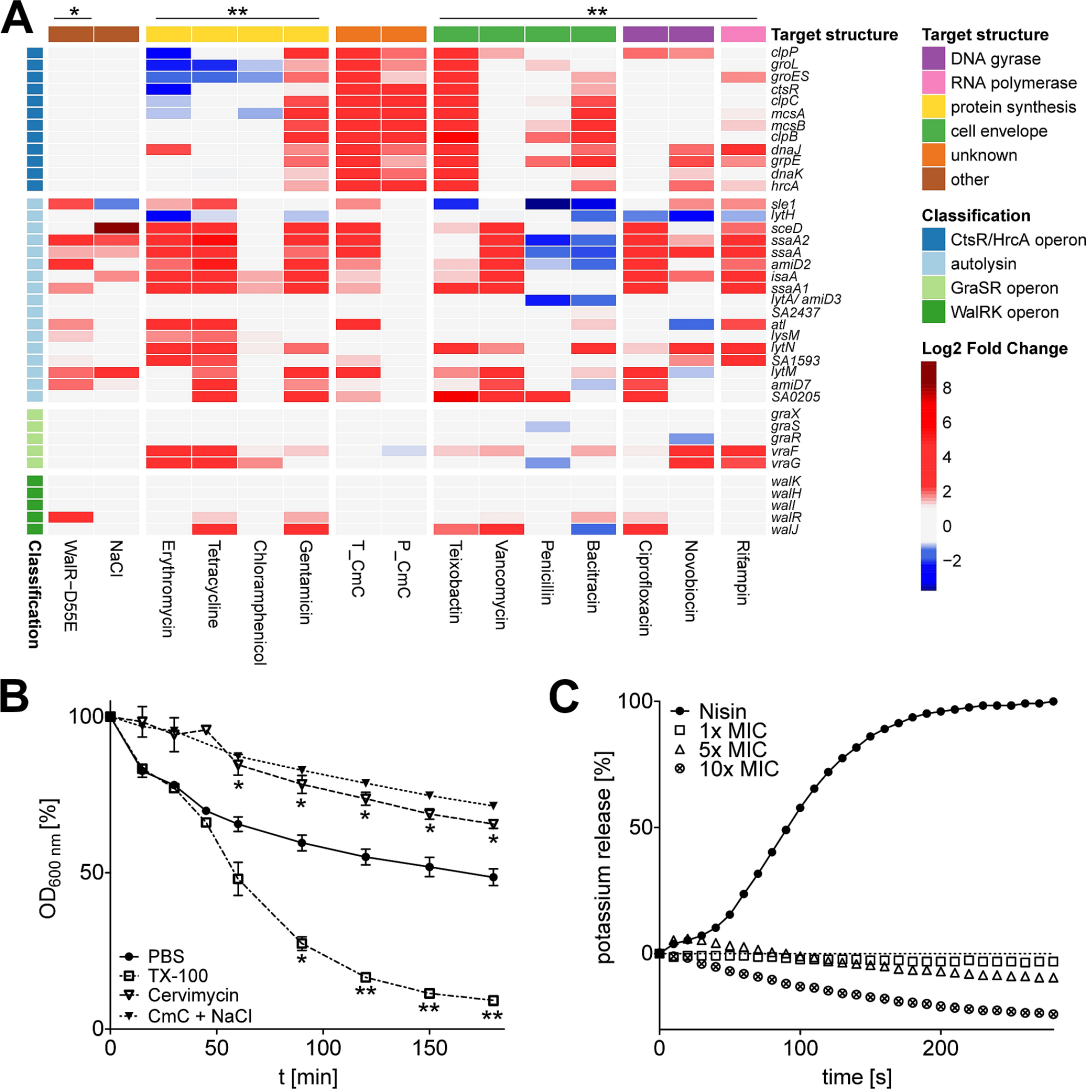
Cervimycin causes a unique induction of the heat shock stress response and autolysin expression, without subsequent cell lysis. Cervimycin induced the expression of the CtsR/HrcA operon and of most autolysins (**A**), a response also seen for other antibiotic classes (comparison with data from Delauné et al. [42] [*] and Jones et al. [40] [**]). Upregulation (log_2_ FC ≥ 1, red color) and downregulation (log_2_ FC ≤ −1, blue color) on the transcriptomic level are compared. T_CmC, cervimycin transcriptome; P_CmC, cervimycin proteome. (**B**) Triton X-100 (TX-100) leads to cell lysis in *S. aureus*, significantly reducing the optical density, while cervimycin treatment prevented cell lysis in the absence and presence of sodium chloride; *P*: *, ≤0.0423, **, ≤0.0059. (**C**) The release of potassium ions by the pore former nisin, but not by cervimycin, confirmed the lack of membrane activity. The graph shows the typical result of one of three experiments.

To obtain a more global view on the effects of cervimycin on the bacterial cell, transcriptomic and proteomic analyses of cervimycin treated versus untreated *S. aureus* SG511 Berlin were performed. In a first attempt, 1 × MIC CmC did not result in a significant regulatory response (data not shown), indicating an inoculum effect. Therefore, exponentially growing *S. aureus* cells were treated with a growth inhibitory concentration of CmC (3 × MIC) for 1 h. After cervimycin treatment, major alterations occurred on a transcriptomic level (564 differentially expressed genes) (Fig. 5A), while minor differences occurred in the proteome (67 differentially abundant proteins) (Fig. 6A; Table S2).

However, neither induction of the SOS response nor elevated expression of the *recF-gyrA-gyrB*, *rib*, and *ure* operons as expected from the gyrase inhibition experiments (Fig. 5B), nor the cell wall stress response as observed with β-lactams, vancomycin, or daptomycin, was detected (Fig. 5C; Table S3).

Increased expression of the *dnaA* and *dnaD* chromosome replication genes, the *dnaN* DNA polymerase III β subunit gene, the *dinB* DNA polymerase IV gene, and the *nth* endonuclease III gene might indicate some DNA stress induced by cervimycin; however, *dnaA* and *dnaD* are also part of the WalR regulon (43). On the other hand, several metabolic pathways were downregulated, like the ArcR, NreC, and Rex regulons, which are crucial for growth under anaerobic conditions (44, 45), the purine biosynthesis genes, parts of the carbohydrate metabolism like the CcpA regulon, the CymR-regulated cysteine metabolism, and major parts of the CodY regulon. Interestingly, the respiratory chain was also partially downregulated, like the *qox* genes, encoding the quinol oxidase, *cydB*, encoding the cytochrome d ubiquinol oxidase subunit, and *ctaAB*, encoding a heme A synthase and a protoheme IX farnesyltransferase, respectively. The SigB-regulon was partially up- and downregulated. The detailed transcriptome data of all genes and proteins differentially expressed in response to CmC are presented in Table S3.

### Cervimycin induces upregulation of most autolysins

Strikingly, the expression of WalR-regulated autolysins increased under cervimycin treatment, which is also seen with gentamicin and other protein biosynthesis inhibitors (Fig. 6A). Most autolysins in *S. aureus* are positively regulated by the WalRK TCS, which becomes apparent when WalR activity is constitutively activated by the phosphomimetic amino acid exchange D55E (42) (Fig. 6A). Except for *lysM*, autolysins regulated by the WalR response regulator (16) were upregulated in the cervimycin-treated samples (Fig. 6A). On a proteomic level, these autolysins were not detected, possibly due to the extracellular localization of mature autolysins. Interestingly, the addition of sodium chloride also increased autolysin transcription (Fig. 6B), and the CmC MIC of *S. aureus* SG511 was decreased by two titer steps to 0.5 µg/mL in the presence of 1 M NaCl. In addition, the overexpression of WalRK caused cell lysis of *S. aureus* in the presence of increasing sodium chloride concentrations (Fig. S5). However, two distinct cell lysis experiments confirmed that induction of autolysis is not a primary effect of cervimycin (Fig. 6B and C).

### Cervimycin induces upregulation of genes indicating protein damage

The modes of action of many antibiotics may be identified by comparing the expression of 69 *S. aureus* genes with well-characterized antibiotics (40). Testing the cervimycin stress response with this model confirmed the clustering of well-known substances according to their mode of action (Fig. S6). Surprisingly, the cervimycin stress response showed the strongest resemblance to protein biosynthesis inhibitors (Fig. S6), but many genes that underwent major transcriptomic alterations due to cervimycin treatment were not included in the 69 gene subset. Comparing the complete transcriptome, cervimycin resembled gentamicin, which induces mistranslation at the ribosome in a concentration-dependent manner (46), causing a strong protein damage response in *S. aureus*, as revealed by induction of the CtsR and HrcA operons. Genes for the production of queuosine-modified tRNAs and tRNA genes in general (43 of 58 genes, 74%) were also de-repressed (Fig. 5A). However, employing an *in vitro* ribosomal toeprinting assay with *E. coli* as well as *B. subtilis* ribosomes with 50 µM CmC (corresponding to 31.25 × MIC of *S. aureus* SG511), we were unable to obtain any evidence that cervimycin inhibits protein synthesis or causes ribosome stalling.

## DISCUSSION

In this mode of action study, we characterized the effects of the polyketide antibiotic CmC on the Gram-positive bacteria *B. subtilis* and *S. aureus*. Both *B. subtilis* and *S. aureus* displayed chromosome and cell division defects after cervimycin treatment. *B. subtilis* cells grew in filaments and chromosomes were condensed and inaccurately distributed and *S. aureus* cells were of uneven size with thickened cell walls.

A filamentation phenotype is quite common in regulatory *Bacillus* mutants, like the *sigD* or *sin* mutant (47), or at elevated growth temperatures (48°C) (48). The heat-induced filamentation of *B. subtilis* was reversed by the addition of *Bacillus* autolysin extracts or lysozyme (48). The cell separation defect of the *Bacillus clpP* deletion mutant is not unicausal, but was at least partially attributed to the accumulation of the ClpP substrate MurAA, which catalyzes the first committed step in the peptidoglycan biosynthesis pathway (49), and might lead to increased cell wall synthesis. Here, the filamentation phenotype of cervimycin-treated *Bacillus* might also indicate missing autolysin activity, which fits well to the cell wall thickening observed in *S. aureus*.

In cervimycin-treated *S. aureus*, most strikingly, septa were misshapen, namely, septa of uneven length, curving, and thinning of the upper part were observed. Furthermore, the nucleoid seemed to impair septum formation. Chromosome replication and segregation are affected by various conditions, such as the lack of SpoIIIE and FtsK DNA translocases, the XerC or RecA recombinases, the RecU resolvase, or in the presence of the gyrase and topoisomerase IV inhibitor nalidixic acid (50). The bacterial DNA gyrase is an essential type II topoisomerase, which is involved in key cellular processes, like DNA replication and alteration of the topology of DNA, in particular the induction of negative supercoils, which necessitates the cleavage of both DNA strands (51). DNA gyrase is composed of the A subunit, which cleaves and re-ligates the DNA, and the B subunit, which is an ATPase. The GyrA-binding fluoroquinolones induce the SOS response in *S. aureus* (26), which induces recombination, DNA repair, and lesion bypass, and modify the transcription of cell-cycle checkpoint proteins (26). On the other hand, the GyrB-binding aminocoumarin novobiocin leads to increased transcription of the *recF-gyrB-gyrA*, *rib*, and *ure* operons, and decreased transcription of *arlRS*, *recA*, *lukA*, *hlgC*, and *fnbA* (30).

Indeed, precursor incorporations tests and a weak signal produced by *Bacillus* reporter strains had suggested a DNA-related target of cervimycin (7, 8), and high cervimycin concentrations inhibited the DNA gyrase supercoiling activity *in vitro*, but the cellular response of *S. aureus* toward cervimycin was not similar to the response to other gyrase-targeting agents (Fig. 5B).

Interestingly, the mode of action of another polyketide antibiotic produced by *Streptomyces coelicolor* M510—actinorhodin—displayed strong similarities to cervimycin, because it also inhibited DNA gyrase with some degree of specificity, caused mutations in the *wal* operon in *S. aureus*, and was more effective at a low pH and only active against Gram-positive bacteria (52). In addition, both cervimycin and actinorhodin induced the protein damage response (CtsR/HrcA operon) and a specific DNA stress response (upregulation of *dinB* and *nth*), but not the LexA-dependent SOS response or the shifts observed in aminocoumarin-treated cells. The authors concluded that actinorhodin most likely acts through oxidative damage to DNA, proteins, and cell envelope (52). However, in contrast to cervimycin, actinorhodin was bacteriostatic and autolysin genes were downregulated under actinorhodin treatment (52), pointing out some major differences between the modes of action of these antibiotics.

Juglone (53) and lapachol (54), which share a quinoid substructure with cervimycin, also cause oxidative stress in *S. aureus.* In addition, juglone is thought to block replication and transcription by binding to DNA (53). For highly or fully substituted quinones, redox-cycling activity rather than thiol alkylation was seen (55). Interestingly, ClpB and McsB from the CtsR operon accumulated in juglone-treated *S. aureus* (53), and lapachol completely de-repressed transcription of the CtsR operon (54). However, lapachol further induced oxidative stress regulons (PerR, HypR, QsrR, MhqR) and cell wall/general stress genes (SigB and GraRS regulons) (54). Such an oxidative stress response was not apparent after cervimycin treatment. The response of the GraS regulon, however, cannot be compared because *S. aureus* SG511 Berlin harbors a truncated GraS kinase (56).

The abovementioned *wal* operon encodes the essential WalRK two-component system, which comprises the WalK kinase and its cognate response regulator WalR, which regulates global autolytic activity, particularly via the bi-functional autolysin AtlA and the endopeptidase LytM. The essentiality of WalRK can be overcome by constitutive expression of *ssaA* or *lytM*, which are two autolysins required for plasticity of the cell wall (57). The system influences peptidoglycan maturation, cell wall turnover, cell separation, protein secretion, and biofilm formation (16). Cell wall biosynthesis and cell wall hydrolysis need to be tightly controlled to allow cell growth (58). An imbalance of these processes, e.g., by vancomycin or methicillin treatment, leads to a lethal cell wall thickening or induces cell lysis, respectively (58). This is underscored by the induction of cell lysis in a sodium chloride-treated WalRK-overexpression strain (Fig. S5). High WalRK levels, as well as high salt concentrations, synergistically increased autolysin transcription (Fig. 6A [59, 60]), leading to cell lysis at the entry into stationary phase. The increased autolysin transcription in cervimycin-treated cells and the synergy of cervimycin and sodium chloride might have indicated a similar process, but, against our expectations, cervimycin combined with sodium chloride inhibited cell lysis in *S. aureus* (Fig. 6B and C).

Recently, a proteomic response library of *B. subtilis* covering 91 antibiotics and comparator compounds of different modes of action was published (61). The authors identified marker proteins, which specifically accumulate due to the impairment of cellular processes and structures (61). Tetracyclines inhibit protein biosynthesis and lead to accumulation of ribosomal proteins like RpsB, RpsF, or RplJ or elongation factor Tu (TufA). In contrast, other antibiotics that target protein biosynthesis (i.e., puromycin, which leads to the premature termination of translation; aminoglycosides, which interfere with ribosomal decoding and proofreading; and acyldepsipeptides, which cause uncontrolled proteolysis by ClpP) elicit the upregulation of the chaperone systems GroEL/GroES and DnaK/DnaJ as well as the proteases ClpC and ClpE in *B. subtilis* (61). These proteins prevent the aggregation of misfolded proteins, facilitate refolding, or aid in the degradation of dysfunctional proteins, and are also highly abundant in cervimycin-treated *S. aureus* (Fig. 6A).

In addition to that, cervimycin induced the transcription of eight ribosomal genes and numerous tRNA genes in *S. aureus* (Table S3; Fig. 5). In a co-culture of *S. aureus* and *Pseudomonas aeruginosa*, increased expression of tRNAs and ribosomal genes in *S. aureus* was attributed to a decrease in translation efficiency (62). However, stalling of the ribosome by cervimycin was not displayed by the *bmrC* (former: *yheI*) bioreporter strain, precursor incorporation tests in *B. subtilis* (7, 8), and ribosome toeprinting. In addition, Sanger sequencing of the ribosomal genes of a cervimycin-resistant mutant (CmR-02) revealed no mutations at these sites. Nevertheless, the test systems used here would be incapable of detecting mistranslation at the ribosome, which, therefore, cannot be excluded for cervimycin. Aminoglycosides induce misreading at relatively low concentrations, 2 × MIC, while much higher concentrations are needed to inhibit protein biosynthesis *in vivo* (35 × MIC) (63, 64). The bactericidal effect of aminoglycosides is thought to rely on faulty membrane proteins causing membrane damage (65). Under salt stress, membrane proteins like the ABC transporter MtsABC are needed to counteract the osmotic stress (59), which might be an alternative explanation for the synergism of cervimycin and sodium chloride, if cervimycin would induce mistranslation at the ribosome or another kind of protein damage.

Electron micrographs of aminoglycoside-treated *S. aureus* resemble cervimycin-treated cells, as cell walls are thickened and rough, and with amikacin also, D-shaped cells occurred (66), indicating a lack of autolysin activity in the presence of cervimycin and amikacin. In this case, the increased autolysin transcription rate suggests that the cells try to compensate a lack of autolysin activity that occurs, e.g., through production of inactive, mistranslated autolysins or problems during transport or processing. The signal of WalK in *S. aureus* is still unclear, but for *B. subtilis*, the cell wall cleavage products of the autolysins CwlO and LytE serve as a signal (67). If the autolysins were not active or not present, such a signal would not be present and WalRK might compensate the perceived lack of autolysins by an increased transcription of the autolysin genes, a process that seems not successful in the presence of cervimycin and might contribute to the protein stress within the cells. The selection of cervimycin-resistant (CmR) *S. aureus* mutants yielded strains with mutations in *walK* that decreased WalK activity (7). This underlines that a functioning WalRK system might play a role in the antibacterial effect of cervimycin and that the absence of WalRK activity might stabilize the cells. However, WalRK is essential in *S. aureus*. Here, the combination of the WalK mutations with loss-of-function mutations in ClpP or ClpC, which were also always present in the resistant mutants (7), may be essential. A *clpP* knockout mutant showed an upregulation of several WalRK-regulated genes, including the essential genes *ssaA* and *lytM* (68). This could provide the necessary baseline concentrations of the essential autolysins in the presence of a low or unresponsive WalK activity in the resistant mutants. On the other hand, recent results show that WalK interlinks cell wall homeostasis and DNA replication, and is the “master regulator of cell growth” (43). In addition, WalK or WalR polymorphisms have been described not only for VISA (32, 33, 36) but also in strains resistant to actinorhodin as described above (52), chlorhexidine (69), siamycin I (70), streptomonomicin (71), cerein 7B, and cerein B4080 (72), indicating that WalK/WalR mutations might also induce slowly growing cells with thick cell walls and concomitant tolerance to antibiotics.

**Fig 7:**
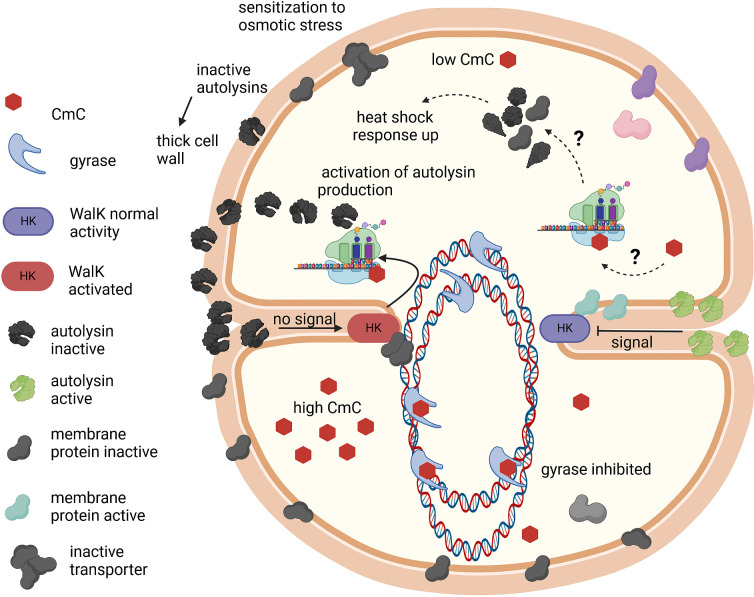
The effects of CmC in *Staphylococcus*. At low CmC concentrations (top), a protein stress response is induced by a so-far unknown mechanism, possibly by mistranslation at the ribosome. The protein stress leads to export of inactive (shown in black) autolysins; this presence of inactive autolysins in the cell wall in turn leads to a thickened peptidoglycan layer and a compensatory further activation of autolysin transcription. Inactivation of membrane proteins involved in chromosome segregation and transport induces the observed chromosome segregation defects; inactivation of membrane proteins involved in osmotic stress sensitizes the cells to high salt concentrations. At very high CmC concentrations (bottom), the bacterial gyrase is inhibited with low affinity. This figure was created with BioRender.com.

In conclusion, cervimycin treatment caused severe structural alterations in *B. subtilis* and *S. aureus*, with cell division, cell wall, and chromosome segregation defects. The hypotheses that explain the phenotypes seen with CmC-treated cells are depicted in Figure 7. Cervimycin decreased the incorporation of radioactively labeled thymidine into the DNA of *B. subtilis* (8), and the DNA gyrase subunit B was inhibited by high concentrations of cervimycin. However, omics analysis revealed a dual de-repression of the heat shock response genes and the autolysin genes in *S. aureus*, while the DNA SOS response or the cell wall stress response was not induced. The expression profiles resembled the stress response toward the aminoglycoside antibiotic gentamicin, indicating a mechanism that leads to protein damage and stress and in which the presence of a functional version of the kinase WalK seems to play a role, as a possible mode of action of cervimycin. In order to further explore the missing links between WalK and protein metabolism, future experiments could monitor the phosphoproteome of treated cells. Ribosomes are switched off via phosphorylation by the serine-threonine kinase PknB (73). This kinase is also involved in phosphorylation of WalR (74, 75) and there is still an unclear connection between low expression of the autolysins SsaA or IsaA and hypersusceptibility to some protein biosynthesis inhibitors (76, 77). Further options would include lipidomics, as the effect of WalK on the membrane composition in *S. aureus* is still unclear (78) and could play a role in the action of cervimycin.

## MATERIALS AND METHODS

### Antimicrobial susceptibility testing

Determination of minimal inhibitory concentrations (MICs) was performed in polystyrene round-bottom microtiter plates (Greiner, Frickenhausen, Germany) using cation-adjusted Müller Hinton (MH) broth. An inoculum of 5 × 10^5^ CFU/mL was employed in the arithmetic broth microdilution method in a final volume of 100 µL/well. The MIC was defined as the lowest concentration of the antibiotic that inhibited visible growth after 24 h incubation at 37°C without agitation. CmC was dissolved in dimethyl sulfoxide (DMSO). The final concentration of DMSO in the MIC assay was below 1%. Bacterial strains and plasmids used in this study are listed in Table S4.

### Time-kill kinetic studies

*B. subtilis* 168 was inoculated into fresh Müller Hinton broth and was grown at 37°C under aeration until it reached an optical density at 600 nm (OD_600_) of ~0.3. Then, 100-µL aliquots of the culture were transferred into a polystyrene round-bottom microtiter plate and were treated with 6 × MIC cervimycin (*Bacillus*: 1.5 µg/mL) or the same amount of DMSO. At defined time-points (0, 1, 5, and 24 h after cervimycin treatment), the cells were serially diluted in 0.9% NaCl and 100 µL was plated on tryptic soy agar (TSA), respectively. The plates were incubated at 37°C overnight, and then the number of colonies was determined.

### Antisense-based susceptibility profiling

The vector-based antisense clones were created in *S. aureus* RN4220 by Forsyth et al. (79), evaluated by Donald et al. (31), and provided by Merck (USA). The pEPSA5 vector is xylose inducible and selected by 34 µg/mL chloramphenicol. The antisense strains were inoculated into lysogeny broth (LB) and were grown at 37°C without shaking. The xylose concentrations (Table 3) employed here were derived from Donald et al. (31), Table S1. Twenty microliters of the cell suspension was diluted in 4 mL 0.9% sodium chloride, poured on LB agar with chloramphenicol and xylose, allowed to settle for 2 min, and then the surplus suspension was removed. The inocula of the antisense strains and the empty vector control were adjusted to yield similar inhibition zones with the control antibiotics. Antibiotic discs with 5 µg ciprofloxacin, 30 µg vancomycin, and 25 µg CmC were added. Inhibition zones were measured after overnight incubation at 37°C. The same procedure was used to measure the effect of protein overexpression on antibiotic susceptibility (plasmids are listed in Table S4).

### Electron microscopy

For scanning transmission electron microscopy (STEM) sample preparation, brain-heart infusion (BHI) was inoculated with 1% of *S. aureus* SG511 pre-culture and was incubated with aeration at 37°C until it reached an OD_600_ of 0.5. Then, the culture was either treated with 3 × MIC CmC (6 µg/mL) or the same amount of DMSO (solvent control) for 1 h. Afterward, sample preparation and image acquisition were performed as described previously (7). The close-up photograph of the untreated control cell was reproduced from Dietrich et al. (7).

### Fluorescence microscopy

A pre-culture of *B. subtilis* 168 was grown in LB at 37°C under shaking overnight, inoculated into fresh LB medium and grown to an OD_600_ of ~0.2 at 37°C, transferred to a polystyrene round-bottom microtiter plate (Greiner, Frickenhausen, Germany), and treated with 8 × MIC CmC (2 µg/mL) or left untreated. *B. subtilis* 168 ∆*clpP* was grown in the presence of 80 µg/mL spectinomycin hydrochloride. Samples of 25 µL were taken at different time-points. Cells were stained with 0.25 mg/mL DAPI in order to visualize DNA and 0.01 mg/mL Nile red (both Sigma-Aldrich, St. Louis, MO, USA) to visualize the cell membrane, and were analyzed by phase-contrast or fluorescence microscopy. To this end, 1-µL samples of the stained cells were placed onto a microscope slide covered with a thin layer of 1% agarose and were analyzed using a Zeiss Axiovert 200M microscope equipped with a Photometrics CoolSnap HQ CCD camera (Roper Scientific). Image acquisition was performed using the microscope software ZEN2011.

### Cloning, overexpression, and purification of *S. aureus* DNA gyrase

The *gyrA* and *gyrB* genes from *S. aureus* SG511 Berlin were amplified using the oligonucleotide primers listed in Table S5, digested with the listed restriction enzymes, cloned into the corresponding sites of the vector pET19 (*gyrA*, Novagen) and pET21 (*gyrB*, Agilent, Santa Clara, CA, USA), and transformed into *E. coli* JM109, respectively. Plasmid integrity was ensured via Sanger sequencing.

*E. coli* BL21 LOBSTR (80) was used for overexpression of the proteins, and the *E. coli* chaperones GroESL were simultaneously expressed from pREP4groESL(MT) (81) to facilitate protein folding. *E. coli* BL21 LOBSTR was grown in terrific broth at 30°C with aeration, protein expression was induced with 1 mM isopropyl β-d-1-thiogalactopyranoside (IPTG) overnight, and for expression of GyrB, the temperature was lowered to 18°C. Cells were harvested and lysed by ultrasonication. Proteins were purified via Ni-NTA affinity chromatography. GyrA and GyrB were dialyzed (100 mM Tris-HCl, pH 7.5, 2 mM EDTA, 100 mM NaCl, 2 mM dithiothreitol [DTT], 10% [vol/vol] glycerol) using Slide-A-Lyzer Dialysis Cassettes (Thermo Fisher Scientific, Waltham, MA, USA), supplemented with glycerol to a final concentration of 50%, and stored at −20°C.

### Inhibition of DNA gyrase supercoiling activity

*E. coli* and *S. aureus* DNA gyrase supercoiling activity was tested using the *E. coli* DNA Gyrase Drug Screening Kit (TG2001G, TopoGEN Inc., Port Orange, FL, USA). Purified *E. coli* DNA gyrase was provided in the kit; *S. aureus* DNA gyrase was cloned and expressed using the pET system as described above. DNA gyrase supercoiling was assayed following the provider’s instructions. The assays were performed in either *E. coli* DNA gyrase supercoiling buffer (35 mM Tris-HCl, pH 7.5, 24 mM KCl, 4 mM MgCl_2_, 2 mM DTT, 1.8 mM spermidine, 6.5% glycerol, 100 mg/mL bovine serum albumin [BSA], and 1 mM ATP) or *S. aureus* DNA gyrase supercoiling buffer (75 mM Tris-HCl, pH 7.5, 30 mM KCl, 7.5 mM MgCl_2_, 7.5 mM DTT, 75 mg/mL BSA, and 2 mM ATP) (82). Potassium glutamate (900 mM) was added to increase *S. aureus* DNA gyrase activity (83). The reaction was performed in a volume of 20 µL, using 2 U DNA gyrase. Different concentrations of antibiotics or the corresponding amount of solvent was added to the reaction mix and pre-incubated at 37°C for 10 min. The reaction was started by addition of 0.125 mg relaxed DNA. The reactions were stopped after 1 h at 37°C by the addition of 5 µL stop buffer (final concentration: 1% SDS, 5% glycerol, and 0.025% bromophenol blue), extracted with 20 µL chloroform:isoamylalcohol (24:1), briefly vortexed, and centrifuged at 13,500 × *g* for 1 min, and the blue aqueous phase was analyzed by gel electrophoresis in a 2% agarose gel in TAE buffer (40 mM Tris-acetate and 0.01 M EDTA, pH 8.3). DNA was stained with 0.5 mg/mL ethidium bromide, visualized with UV light, and photo documented. The strength of the bands was quantified using the Gel Analyzer 2010a image analysis software.

### Transcriptomics and proteomics

Transcriptomics and proteomics were mainly performed as described earlier (7). Briefly, cultures of *S. aureus* SG511 were grown in tryptic soy broth (TSB) at 37°C with aeration until an OD_600_ of 0.5 was reached, and were treated with 3 × MIC cervimycin C or the same volume of DMSO for 1 h. Salt-treated *S. aureus* was grown in TSB supplemented with 1 M NaCl. The RNA extraction was performed as previously described (7).

After checking the RNA quality via agarose gel electrophoresis, total RNA of cervimycin-treated cells was submitted to the Genewiz sequencing facility (Leipzig, Germany) for ribosomal RNA depletion, library preparation, and strand-specific total RNA sequencing on an Illumina NovaSeq platform (2 × 150 bp sequencing, 10 M read pairs). Raw sequence data generated from Illumina NovaSeq were converted into FASTQ files and de-multiplexed using Illumina bcl2fastq program version 2.20. One mismatch was allowed for index sequence identification. After investigating the quality of the raw data, sequence reads were trimmed to remove possible adapter sequences and nucleotides with poor quality using Trimmomatic v.0.36. The trimmed reads were mapped to the reference genome (*S. aureus* SG511 Berlin; NCBI Reference Sequence: NZ_CP076660.1) using the Bowtie2 aligner v.2.2.6, and binary alignment map (BAM) files were generated as a result of this step. Unique gene hit counts were calculated using featureCounts from the Subread package v.1.5.2. Only unique reads that fell within gene regions were counted. Using DESeq2, a comparison of gene expression between the groups of samples (untreated versus cervimycin treated) was performed. The Wald test was used to generate *P* values and log_2_ fold changes (FCs). Genes with an adjusted *P* value < 0.05 and absolute log_2_ fold change > 1 were listed as differentially expressed genes in the presence of cervimycin. For the NaCl-treated cells, this procedure was performed as described by Dietrich et al. (7). Protein extraction and proteomics were also performed as previously described (7).

### Triton X-100/cervimycin-induced autolysis of *S. aureus*

TSB medium or TSB medium supplemented with 1 M NaCl was inoculated with 1% *S. aureus* SG511 pre-culture and was grown at 37°C with aeration until it reached the exponential growth phase (OD_600_ ~0.6 to 0.8). Then, 1 mL of the culture was harvested (1,844 × *g*, 3 min), washed with 1 mL ice-cold ultrapure water, resuspended in 1 mL phosphate-buffered saline (PBS), and transferred into a polypropylene cuvette, and the initial OD_600_ was determined (=100%). Afterward, CmC (3 × MIC, 6 µg/mL) or the same volume of DMSO was added, and the cuvettes were sealed with parafilm and inverted three to six times. The cuvettes were incubated at 37°C, and the OD_600_ was determined every 15 to 30 min after inverting the cuvettes three to six times. As a positive control, Triton X-100 was used (0.1% final concentration), which is thought to remove lipoteichoic acids from the cell envelope, triggering autolysis (84). Lysis curves were created with Microsoft Excel 2010. Results were normalized to the starting OD_600_.

### Potassium efflux from *S. aureus* cells

TSB medium was inoculated with 3% of an *S. aureus* SG511 pre-culture and was grown at 37°C with aeration until it reached the mid-exponential growth phase (OD_600_ ~1 to 1.5). Cells were harvested (2,254 × *g*, 3 min, 4°C) and washed with 25 mL pre-cooled assay buffer (300 mM choline chloride, 30 mM 2-(N-morpholino)ethanesulfonic acid monohydrate, 20 mM Tris, pH 6.5), and the optical density at 600 nm in assay buffer was adjusted to 30. The perfectION Combination Potassium Electrodes (Mettler-Toledo, Greifensee, Switzerland) were equilibrated in assay buffer and calibrated in standard KCl solutions (1 mM, 0.1 mM, and 0.01 mM in assay buffer) for 40 s each, beginning with the lowest KCl concentration. For sample measurement, 200 µL of *S. aureus* cells were diluted in 1.8 mL assay buffer, and the baseline was measured for 3 min. Then, the 1 ×, 5 ×, or 10 × MIC of CmC (MIC = 2 µg/mL) was added, and the potassium efflux was measured for 5 min. The addition of 1 µM nisin served as a positive control and was assumed to release 100% of the intracellular potassium. Potassium efflux was calculated as a ratio of the measured free potassium and the amount of potassium released by the addition of nisin. The baseline potassium concentration at the beginning of the experiment was subtracted.

### Determination of growth curves

To investigate the effect of WalRK overexpression on the growth behavior, *S. aureus* HG003 pTXvicRK (85) and an empty vector control were grown overnight in BHI medium. *S. aureus* strains harboring the pTX vector, which comprises a xylose-inducible promoter, or its derivatives were grown in the presence of 12.5 µg/mL tetracycline, and 50 mM of xylose was routinely used to induce overexpression from the plasmid. Growth curves were measured in either BHI medium or BHI medium supplemented with sodium chloride, and under un-induced (0 mM xylose) or induced (50 mM xylose) conditions. To this end, *S. aureus* overnight cultures were diluted in the abovementioned media to an optical density at 600 nm wavelength of 0.05 and were transferred into polystyrene round-bottom microtiter plates. Cells were grown at 25°C in a Tecan Infinite M Plex multimode plate reader with aeration, and the OD_600_ was measured every 20 min.

### Sanger sequencing of 23S rRNA genes

The 23S rRNA genes from *S. aureus* CmR-02 were amplified and sequenced using the oligonucleotide primers listed in Table S5.

### Ribosome toeprinting assay

The assay detects the translation arrest of the ribosome in the presence of antibiotics. It uses primer extension inhibition analysis, i.e., reverse transcriptase and a labeled primer are added to an *in vitro* translation assay. cDNA transcription stops when the progress of the reverse transcriptase is impeded by the ribosome stalled on the mRNA. The cDNA fragments were analyzed by SDS gel electrophoresis (86). The assay was performed with 50 µM cervimycin C and *E. coli* as well as *B. subtilis* ribosomes as described by Orelle et al. (86).

## Data Availability

The high-throughput RNA-sequencing data in this publication have been deposited in NCBI’s Gene Expression Omnibus (87) and are available under GEO SuperSeries GSE250541 (GEO data sets GSE209674 and GSE250540). The mass spectrometry proteomics data have been deposited at the ProteomeXchange Consortium via the PRIDE (88) partner repository with the data set identifier PXD034970.

## SUPPLEMENTAL MATERIAL

Supplemental Figures and Tables msphere.00764-23-s0001.pdf Figure S1–S6 and Table S1–S5.

ASM does not own the copyrights to Supplemental Material that may be linked to, or accessed through, an article. The authors have granted ASM a non-exclusive, world-wide license to publish the Supplemental Material files. Please contact the corresponding author directly for reuse.
