# Supplementary material for: The unusual mode of action of the polyketide glycoside antibiotic cervimycin C
Source: mSphere. 2024 May 9;9(5):e00764-23. doi: 10.1128/msphere.00764-23 (PMC11237698; doi:10.1128/msphere.00764-23)
Supplement: Supplemental Figures and Tables — Figure S1–S6 and Table S1–S5. [file msphere.00764-23-s0001.pdf]

Supplementary Data

Figures

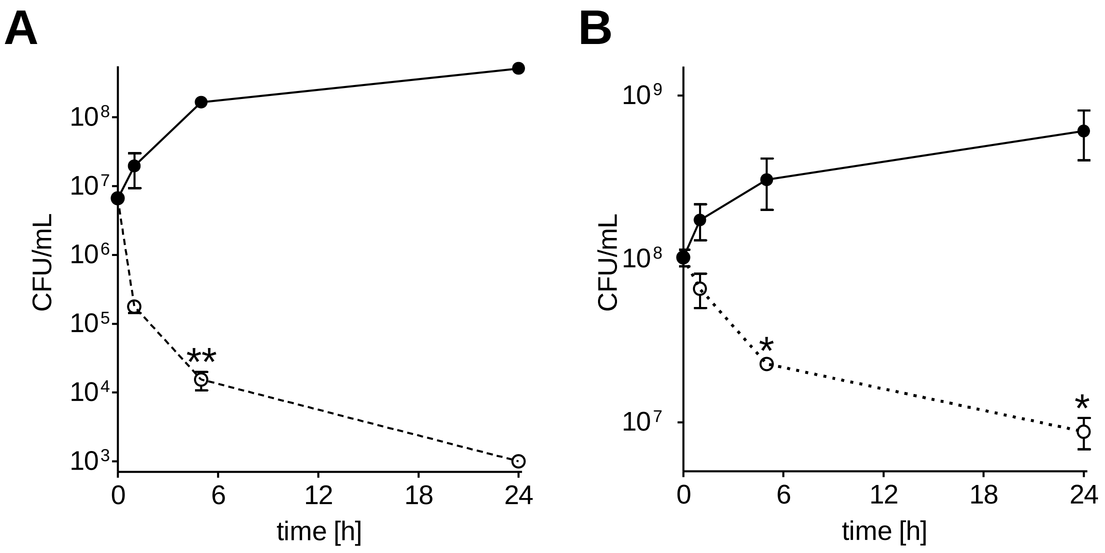

Figure S1: Cervimycin C (CmC) kills Gram-positive bacteria. Exponentially growing *Bacillus subtilis* 168 (A) or *S. aureus* SG511 Berlin (B) were treated with 6 × MIC CmC, respectively, or the same volume of DMSO (untreated), and dilutions of the cultures were plated on TSA after the indicated periods of time; **, p ≤ 0.0055; CFU, colony forming units.

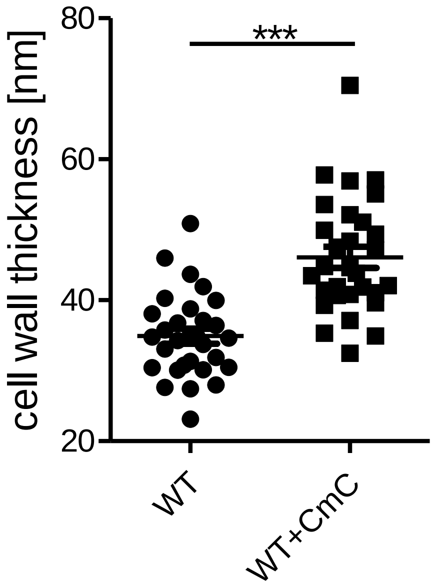

Figure S2: Cervimycin treatment of *S. aureus* SG511 Berlin (WT) leads to a significant increase in cell wall thickness. *S. aureus* was grown in BHI medium and after reaching the exponential growth phase treated with 3 × MIC CmC (6 µg/mL) for 1 h (WT+CmC) or left untreated (WT). Cells were fixed and visualized via scanning transmission electron microscopy. Analysis of the cell wall thickness using the ImageJ software (1) and 100 cells per sample revealed a significant increase in cell wall thickness after cervimycin treatment; ***, $p < 0.0001$.

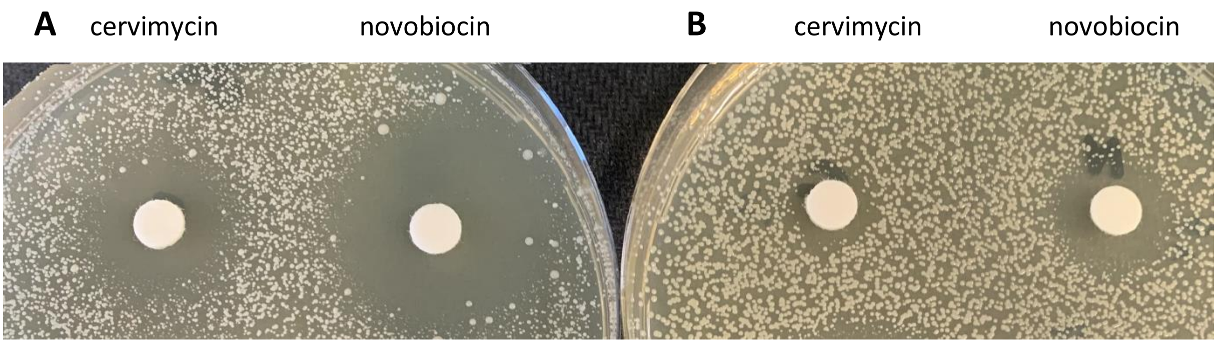

Figure S3: Suppression of growth of the *gyrB* antisense clone in the presence of cervimycin and novobiocin (positive control). Blank discs were loaded with cervimycin (25 µg, left side) and novobiocin (175 ng, right side) and cells were incubated on LB agar (34 mg/L chloramphenicol and 17 mM xylose in A) and 34 mg/l chloramphenicol in B) at 37 °C. In the presence of xylose (A) growth is much slower, and the inhibition zones are enlarged (1.45-fold for cervimycin and 1.8-fold for novobiocin). Larger colonies within the inhibition zones appeared regularly and plasmid sequencing showed that these colonies had mutations in the promoter that drives transcription of the antisense RNA.

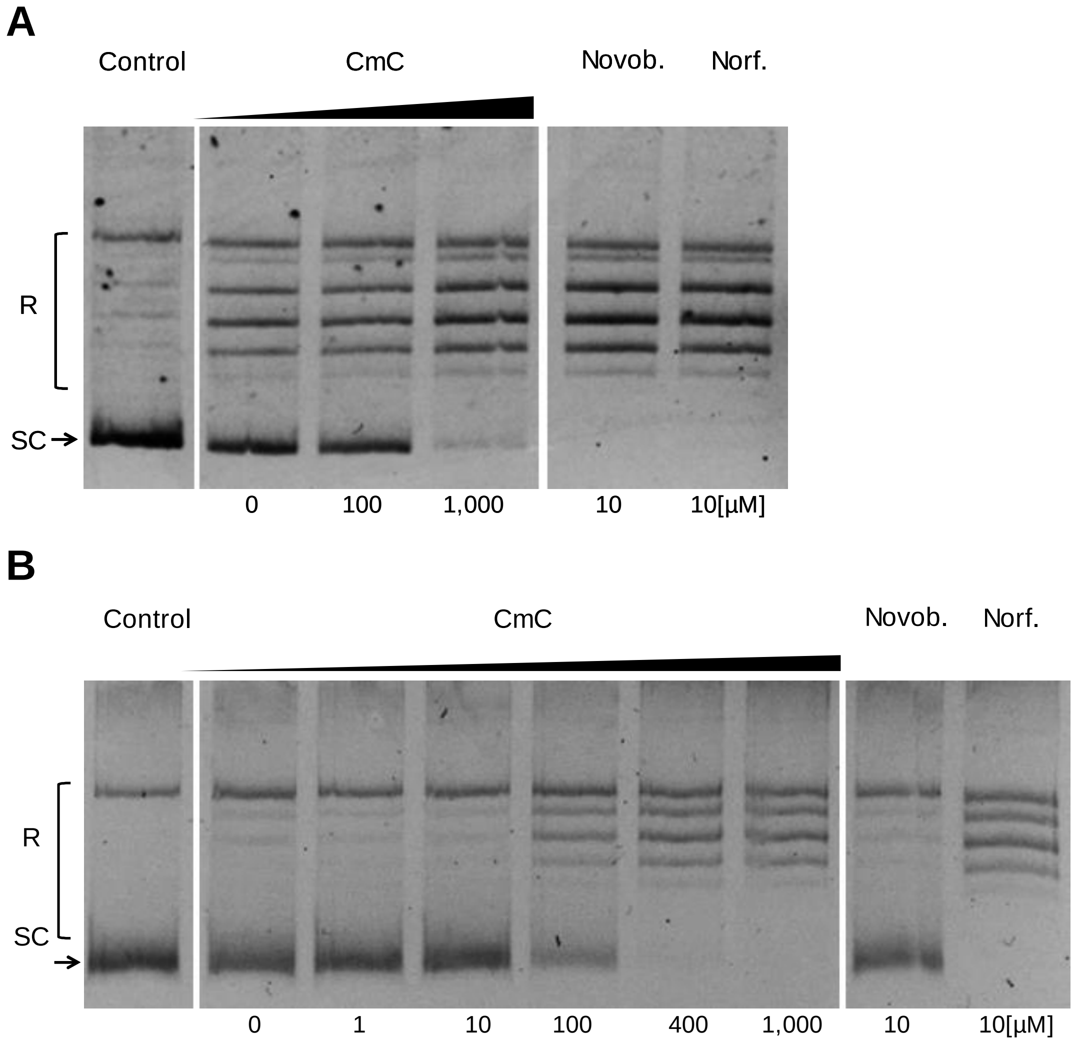

Figure S4: DNA gyrase supercoiling activity. *E. coli* (**A**) and *S. aureus* (**B**) DNA gyrase activity was measured as proportion of supercoiled DNA after incubation of 2 U DNA gyrase with 0.125 mg relaxed plasmid DNA and cervimycin (CmC) for 1 h at 37 °C in comparison to a control (control) treated with the same amount of DMSO. DNA gyrase targeting antibiotics novobiocin (Novob.) and norfloxacin (Norf.) served as positive controls for DNA gyrase inhibition.

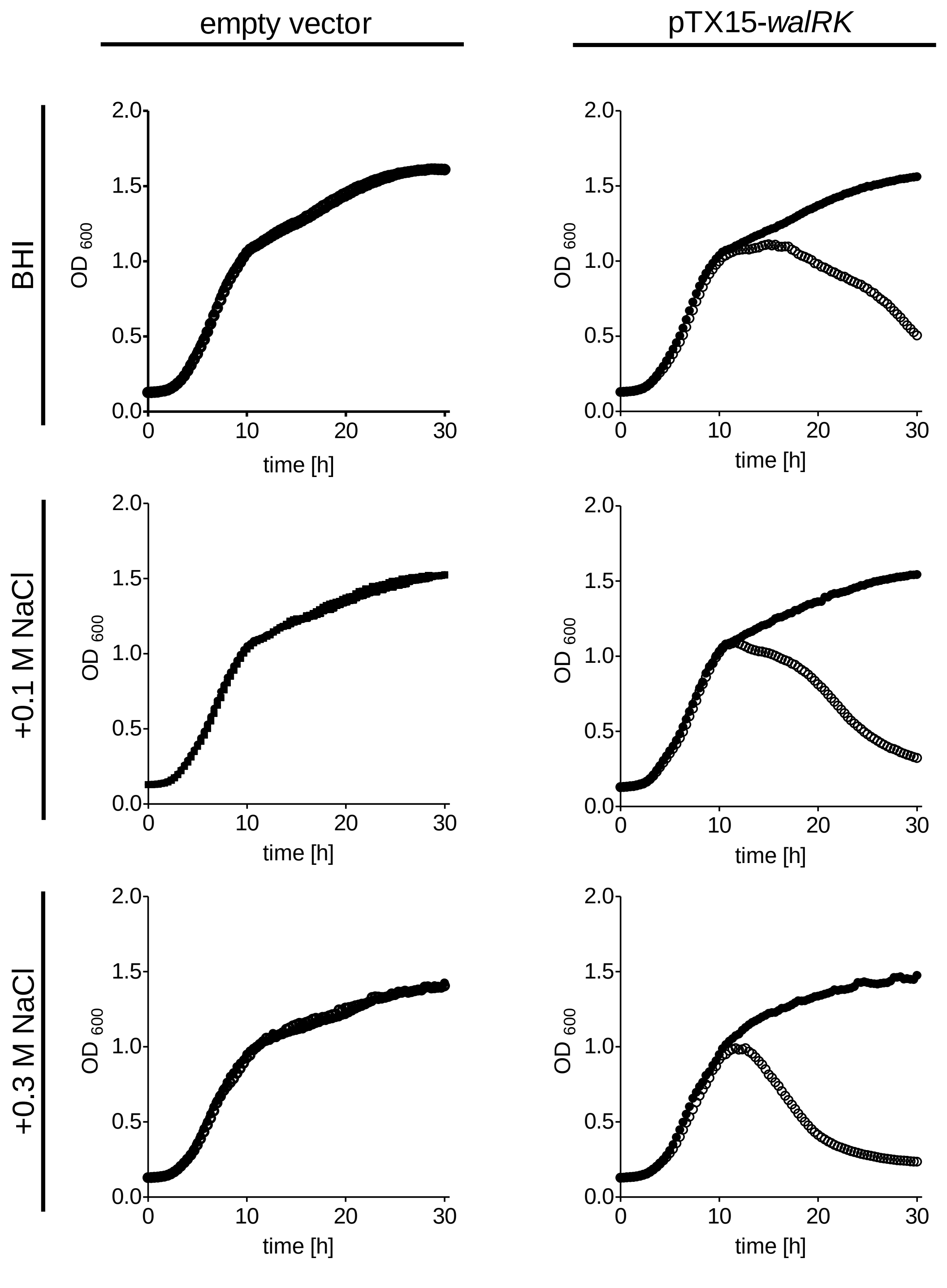

Figure S5: Growth curves of *S. aureus* HG003 pTX15 (empty vector) and *S. aureus* HG003 pTX15-*walRK* in BHI medium with increasing concentrations of sodium chloride. *S. aureus* strains were grown in BHI broth at 25 °C. Overexpression of WalRK was induced by the addition of 50 mM xylose (open circles), or measured without the addition of xylose (closed symbols). Increasing sodium chloride concentrations caused cell lysis of the WalRK overexpression strain at 25 °C, but not of the empty vector control.

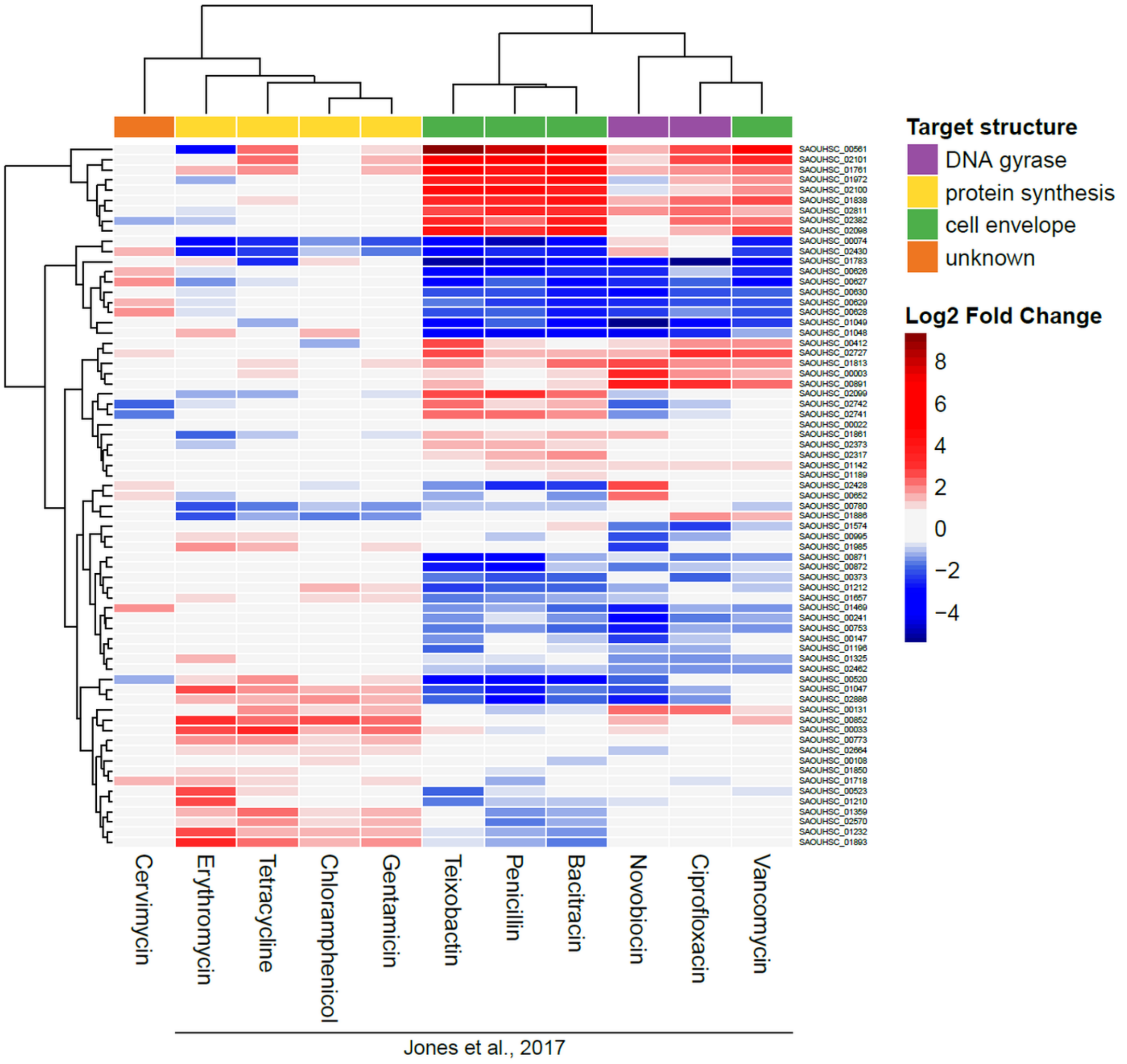

Figure S6: Heatmap of expression levels of 69 *S. aureus* genes predictive of antibiotic mode of action. The heatmap was generated from the data of Jones et al. (2017) (2) and the cervimycin C RNA-Seq experiment (this study) using the ggplot2 R package (3). Differential expression is displayed as Log_2 ratios generated from RPKM values for antibiotic and 2% DMSO-treated *S. aureus* cells. Only significant expression levels were included (*p*-value ≤0.05). Red shading indicates higher expression of *S. aureus* genes compared to the 2% DMSO treatment, blue shading indicates lower expression, grey shading indicates a non-significant expression level/a non-significant alteration (*p*-value > 0.05).

Tables

Table S1: Ciprofloxacin- and novobiocin-resistance mediating gyrase mutations have no effect on cervimycin susceptibility. Antibiotic susceptibility was tested in agar diffusion assays; VA, vancomycin (30 µg); CIP, ciprofloxacin (5 µg); Novo, novobiocin (25 µg); CmC, cervimycin C (25 µg).

	Diameter inhibition zone [mm]			
**Strain**	VA	CIP	Novo	CmC
S. aureus RN4220	16	22	23	11
S. aureus RN4220 $GyrA^{S84L}$	16	**9**	23	11
S. aureus RN4220 $GyrB^{G86S}$	16	22	**17**	11

Table S2: Differential expression/translation of genes/proteins in *S. aureus* SG511 Berlin treated with 3 × MIC cervimycin C in comparison to untreated *S. aureus* SG511 Berlin. Shown is the total number of genes/proteins with a significant detection level (total, p-value ≤0.05), as well as the number of significantly up-regulated (up, Log_2 Fold Change ≥ 1) and down-regulated (down, Log_2 Fold Change ≤ −1) genes/proteins.

Omics	total	diff. expressed	up	down
**transcriptome**	1210	564<br>(46.61%)	298<br>(24.63%)	266<br>(21.98%)
**proteome**	336	67<br>(19.94%)	32<br>(9.52%)	35<br>(10.42%)
**transcriptome and proteome**	1279	25<br>(1.96%)	15<br>(1.17%)	10<br>(0.78%)

Table S3: Transcriptomic and proteomic data of cervimycin treated *S. aureus* SG511 Berlin. Genes/proteins with at least 2-fold differential expression in comparison to the untreated *S. aureus* SG511 and significant p-value ($p \leq 0.05$) are shown, which corresponds to a $\log_2$ Fold Change ≥ 1 or ≤ -1. The specified regulators correspond to the genome of *S. aureus* NCTC 8325 and were derived from the Aureowiki database (4). Significantly differentially expressed genes/proteins are marked in blue (down-regulated) and red (up-regulated), respectively. 0 indicates a non-significant expression/detection level.

						Log_2 Fold Change	
Locus tag KQU62_	Gene Name	Regulator 1	Reg. 2	Reg. 3	Reg. 4	transcriptome	proteome
_00020	-	0	0	0	0	-1.587	0
_00050	-	0	0	0	0	-1.36	0
_00065	-	0	0	0	0	-1.461	0
_00270	-	0	0	0	0	1.641	0
_00390	-	0	0	0	0	1.348	0
_00400	-	0	0	0	0	1.613	0
_00475	-	0	0	0	0	-1.225	0
_00480	-	0	0	0	0	-1.161	0
_00485	-	0	0	0	0	-1.296	0
_00520	-	0	0	0	0	1.491	0
_00635	-	0	0	0	0	1.287	0
_00750	-	0	0	0	0	-1.068	0
_00870	-	0	0	0	0	0	1.38
_00885	-	0	0	0	0	1.214	0
_00935	-	0	0	0	0	-0.628	2.26
_00995	-	0	0	0	0	0	-1.37
_01445	-	0	0	0	0	1.062	0.294
_01510	-	0	0	0	0	1.501	0
_01615	-	0	0	0	0	2.259	0.38
_01620	-	0	0	0	0	2.699	0
_01630	-	0	0	0	0	-1.055	-2.27
_01635	-	0	0	0	0	-1.296	0
_01725	-	0	0	0	0	-1.543	-0.806
_01805	-	0	0	0	0	1.099	0
_01810	-	0	0	0	0	1.037	0
_02010	-	0	0	0	0	0	-1.54
_02030	-	0	0	0	0	1.016	0
_02150	-	0	0	0	0	2.122	0
_02215	-	0	0	0	0	1.28	0
_02270	-	0	0	0	0	1.068	0
_02525	-	0	0	0	0	1.057	0
_02600	-	0	0	0	0	1.793	0

Locus tag KQU62_	Gene Name	Regulator 1	Reg. 2	Reg. 3	Reg. 4	Log2 Fold Change transcriptome	Log2 Fold Change proteome
_02610	-	0	0	0	0	3.066	0
_02620	-	0	0	0	0	3.816	0
_02625	-	0	0	0	0	2.619	0
_02630	-	0	0	0	0	3.168	0
_02635	-	0	0	0	0	2.466	0
_02640	-	0	0	0	0	2.425	0
_02645	-	0	0	0	0	2.329	0
_02650	-	0	0	0	0	1.938	0
_02710	-	0	0	0	0	-2.037	0
_02805	-	0	0	0	0	-1.061	0
_02840	-	0	0	0	0	-1.02	0
_02925	-	0	0	0	0	1.056	0
_03015	-	0	0	0	0	1.086	0
_03165	-	0	0	0	0	1.582	0
_03200	-	0	0	0	0	2.537	0
_03495	-	0	0	0	0	1.287	0
_03500	-	0	0	0	0	1.089	0
_03510	-	0	0	0	0	1.628	0
_03515	-	0	0	0	0	2.192	0
_03520	-	0	0	0	0	1.547	0
_03570	-	0	0	0	0	1.993	0.404
_03775	-	0	0	0	0	0	1.37
_03840	-	0	0	0	0	1.18	0
_03900	-	0	0	0	0	0.253	1.92
_03910	-	0	0	0	0	1.515	0
_03985	-	0	0	0	0	1.128	0
_04065	-	0	0	0	0	-1.069	0
_04460	-	0	0	0	0	-1.369	-0.324
_04795	-	0	0	0	0	1.16	0
_04850	-	0	0	0	0	1.564	0.249
_04855	-	0	0	0	0	1.368	0
_04950	-	0	0	0	0	1.629	0
_05745	-	0	0	0	0	1.688	0
_05885	-	0	0	0	0	1.41	0.405
_06525	-	0	0	0	0	1.395	0
_06570	-	0	0	0	0	1.078	0
_06575	-	0	0	0	0	1.319	0
_06580	-	0	0	0	0	1.527	0
_06610	-	0	0	0	0	1.691	0
_06625	-	0	0	0	0	1.181	0
_06655	-	0	0	0	0	1.178	0
_07055	-	0	0	0	0	1.025	0.24
_07260	-	0	0	0	0	0.622	1.26
_07380	-	0	0	0	0	1.329	0.254

						Log$_2$ Fold Change	
Locus tag KQU62_	Gene Name	Regulator 1	Reg. 2	Reg. 3	Reg. 4	transcriptome	proteome
_07485	-	0	0	0	0	-1.327	0
_07490	-	0	0	0	0	-1.254	0
_07725	-	0	0	0	0	0.294	1.03
_07795	-	0	0	0	0	-1.067	-0.344
_08110	-	0	0	0	0	-1.097	-0.391
_08170	-	0	0	0	0	1.603	0
_08200	-	0	0	0	0	1.2	-0.873
_08210	-	0	0	0	0	1.577	0
_08770	-	0	0	0	0	-1.477	0
_08885	-	0	0	0	0	-0.545	-1.52
_08960	-	0	0	0	0	1.888	0
_09415	-	0	0	0	0	-1.079	0
_09425	-	0	0	0	0	1.32	0
_09580	-	0	0	0	0	-1.155	0
_09655	-	0	0	0	0	1.065	0
_09965	-	0	0	0	0	1.297	0
_09995	-	0	0	0	0	1.346	0
_10050	-	0	0	0	0	1.715	0
_10225	-	0	0	0	0	-1.447	0
_10325	-	0	0	0	0	0	1.19
_10415	-	0	0	0	0	-1.59	0
_10420	-	0	0	0	0	-1.319	-0.431
_10430	-	0	0	0	0	-1.893	-0.503
_10480	-	0	0	0	0	1.253	0.58
_10550	-	0	0	0	0	1.086	0
_10555	-	0	0	0	0	-2.583	0
_10560	-	0	0	0	0	-1.696	0
_10575	-	0	0	0	0	0	-1.43
_10615	-	0	0	0	0	-1.54	0
_10755	-	0	0	0	0	-1.111	-0.259
_10925	-	0	0	0	0	1.203	0
_10930	-	0	0	0	0	1.779	0
_10990	-	0	0	0	0	1.899	0
_11030	-	0	0	0	0	1.263	0
_11045	-	0	0	0	0	-1.902	0
_11115	-	0	0	0	0	-1.299	0
_11125	-	0	0	0	0	1.962	0
_11130	-	0	0	0	0	1.472	0
_11190	-	0	0	0	0	1.007	0
_11255	-	0	0	0	0	-3.335	0
_11260	-	0	0	0	0	-3.373	0
_11285	-	0	0	0	0	-1.14	0
_11310	-	0	0	0	0	1.107	0
_11630	-	0	0	0	0	-2.189	-0.807

						Log$_2$ Fold Change	
Locus tag KQU62_	Gene Name	Regulator 1	Reg. 2	Reg. 3	Reg. 4	transcriptome	proteome
_11635	-	0	0	0	0	-1.835	0
_11640	-	0	0	0	0	-1.452	0
_11750	-	0	0	0	0	2.71	0
_11805	-	0	0	0	0	1.399	0
_12165	-	0	0	0	0	-1.621	0
_12180	-	0	0	0	0	1.62	0
_12200	-	0	0	0	0	-1.275	0
_12250	-	0	0	0	0	-1.512	0
_12255	-	0	0	0	0	-1.453	0
_12260	-	0	0	0	0	-1.189	0
_12275	-	0	0	0	0	-1.749	0
_12280	-	0	0	0	0	-1.537	0
_12505	-	0	0	0	0	-1.634	0
_12555	-	0	0	0	0	-1.176	0
_12610	-	0	0	0	0	2.745	0
_12615	-	0	0	0	0	1.605	0
_12850	-	0	0	0	0	-1.799	0
_12895	-	0	0	0	0	1.554	0
_12975	-	0	0	0	0	-1.242	0
_13035	-	0	0	0	0	-1.951	0
_13040	-	0	0	0	0	-2.472	0
_13050	-	0	0	0	0	-1.813	0
_13175	-	0	0	0	0	-2.082	0
_13200	-	0	0	0	0	2.664	0
_13250	-	0	0	0	0	-1.278	0
_13255	-	0	0	0	0	-1.137	0
_13265	-	0	0	0	0	-1.939	-0.475
_13270	-	0	0	0	0	-1.379	0
_13330	-	0	0	0	0	-1.061	0
_13335	-	0	0	0	0	-1.158	0
_13345	-	0	0	0	0	-1.242	0
_13350	-	0	0	0	0	-1.2	0
_13360	-	0	0	0	0	-1.572	0
_13365	-	0	0	0	0	-1.483	0
_13370	-	0	0	0	0	-1.654	0
_13375	-	0	0	0	0	-1.211	0
_13380	-	0	0	0	0	-1.528	0
_13385	-	0	0	0	0	-1.569	0
_13400	-	0	0	0	0	-1.372	0
_13405	-	0	0	0	0	-2.312	0
_13415	-	0	0	0	0	-1.5	0
_01225	23S rRNA	0	0	0	0	1.581	0
_08005	23S rRNA	0	0	0	0	1.177	0
_08970	23S rRNA	0	0	0	0	1.047	0

						Log$_2$ Fold Change	
Locus tag KQU62_	Gene Name	Regulator 1	Reg. 2	Reg. 3	Reg. 4	transcriptome	proteome
_05505	acyP	0	0	0	0	1.055	0
_08115	ampS	0	0	0	0	-1.174	-0.295
_12000	asp1	0	0	0	0	-1.46	-1.97
_11990	asp3	0	0	0	0	-1.46	0
_12360	azlC	0	0	0	0	-1.256	0
_12365	azlD	0	0	0	0	-1.647	0
_12890	azoR	0	0	0	0	1.018	0
_11755	betA	0	0	0	0	3.033	1.23
_11760	betB	0	0	0	0	2.822	0
_04280	bshC	0	0	0	0	1.292	0
_12530	cap8H	0	0	0	0	1.2	0
_09815	cbiO	0	0	0	0	0.395	-1.53
_11410	cidA	0	0	0	0	1.111	0
_06970	citZ	0	0	0	0	1.221	0.408
_11085	cntA	0	0	0	0	-1.175	0
_06790	comC	0	0	0	0	1.552	0
_06455	comEC	0	0	0	0	1.494	0
_06240	comGA	0	0	0	0	1.039	0
_06235	comGB	0	0	0	0	1.204	0
_10630	corA2	0	0	0	0	1.047	0.938
_07425	crcB1	0	0	0	0	1.912	0
_09085	cshA	0	0	0	0	1.291	0
_03955	ctaA	0	0	0	0	-1.924	0
_03960	ctaB	0	0	0	0	-1.624	0
_03965	ctaM	0	0	0	0	-1.531	0
_11770	cudC	0	0	0	0	1.395	0
_11775	cudT	0	0	0	0	2.682	0
_03810	cydB	0	0	0	0	-1.066	0
_11810	cysG	0	0	0	0	1.049	0
_11815	cysJ	0	0	0	0	1.103	0.935
_06050	cysM	0	0	0	0	1.295	0
_01435	dgk	0	0	0	0	0	1.6
_08205	dinB	0	0	0	0	1.616	0
_04315	divIB	0	0	0	0	-0.302	1.71
_02985	dltB	0	0	0	0	-0.396	-1.24
_12315	dnaA	0	0	0	0	1.629	-0.311
_05755	dnaD	0	0	0	0	1.921	0
_12320	dnaN	0	0	0	0	1.186	0.229
_03225	eap	0	0	0	0	-1.263	0
_10475	emrB	0	0	0	0	1.298	0.525
_13275	esxA	0	0	0	0	-1.943	0
_05910	fer	0	0	0	0	2.668	0
_10785	fmhA	0	0	0	0	-1.016	-0.311
_03655	fmtA	0	0	0	0	1.392	0

						Log$_2$ Fold Change	
**Locus tag KQU62_**	**Gene Name**	**Regulator 1**	**Reg. 2**	**Reg. 3**	**Reg. 4**	**transcriptome**	**proteome**
_07830	fumC	0	0	0	0	-1.014	0
_08240	gatC	0	0	0	0	2.172	0
_09990	glcU	0	0	0	0	1.294	0
_04370	gloA2	0	0	0	0	1.309	0
_02795	gpmA1	0	0	0	0	1.278	0
_06435	hemW	0	0	0	0	1.03	0
_13045	hmp	0	0	0	0	-2.317	0
_06450	holA	0	0	0	0	1.285	0
_12955	hptA	0	0	0	0	-1.009	0
_12950	hptS	0	0	0	0	-1.047	0
_00690	hsdM1	0	0	0	0	-0.493	3.85
_12855	hsdR	0	0	0	0	-1.468	-0.569
_01135	hslO	0	0	0	0	1.524	0.479
_10675	hsp20	0	0	0	0	-2.358	0
_06965	icd	0	0	0	0	1.066	0
_02605	integrase	0	0	0	0	1.154	0
_10605	iruO	0	0	0	0	1.293	0
_09075	kdpD	0	0	0	0	-1.04	0
_09080	kdpE	0	0	0	0	-1.144	0
_09695	lacR	0	0	0	0	-1.006	0
_11705	ldh2	0	0	0	0	-2.047	0
_03205	leuA2	0	0	0	0	2.075	0
_00720	lpl1	0	0	0	0	-1.064	0
_00715	lpl5	0	0	0	0	-1.334	0
_00745	lpl7	0	0	0	0	-1.198	0
_13165	lrgA	0	0	0	0	-6.163	0
_13170	lrgB	0	0	0	0	-6.684	0
_13160	lytR	0	0	0	0	0.687	-1.71
_06040	malR	0	0	0	0	-1.259	0
_09395	manA	0	0	0	0	0.241	-2.08
_03585	menD	0	0	0	0	1.386	0
_07475	menE	0	0	0	0	0.409	-1.56
_03580	menF	0	0	0	0	1.613	0.513
_03590	menH	0	0	0	0	1.372	0
_00210	mepB	0	0	0	0	2.389	0
_00310	metE	0	0	0	0	-1.792	-0.795
_11300	mhqR	0	0	0	0	1.118	0
_03050	mnhG	0	0	0	0	0	1.86
_13230	mnmA	0	0	0	0	-1.648	-0.394
_10110	modA	0	0	0	0	-1.007	0
_10105	modB	0	0	0	0	-1.288	0
_10100	modC	0	0	0	0	-1.216	0
_00800	mpsB	0	0	0	0	1.018	-0.504
_05490	msa	0	0	0	0	1.147	0

						Log$_2$ Fold Change	
Locus tag KQU62_	Gene Name	Regulator 1	Reg. 2	Reg. 3	Reg. 4	transcriptome	proteome
_05500	msaA	0	0	0	0	1.299	0
_08050	msbA	0	0	0	0	1.415	0
_03000	nfu	0	0	0	0	1.189	0
_05670	norB	0	0	0	0	-2.852	0
_05750	nth	0	0	0	0	1.777	1.29
_07105	ohr	0	0	0	0	1.051	0
_13030	opp-5A	0	0	0	0	-2.198	0.629
_02235	pabA	0	0	0	0	1.02	0
_01235	pdxR	0	0	0	0	1.73	0
_06165	pepQ2	0	0	0	0	1.039	0
_11180	pgcA	0	0	0	0	1.266	0
_08460	phage-related	0	0	0	0	2.136	0
_08475	phage-rel.	0	0	0	0	1.053	0
_08600	phage-rel.	0	0	0	0	1.405	0
_08615	phage-rel.	0	0	0	0	2.087	0
_08620	phage-rel.	0	0	0	0	2.159	0
_08625	phage-rel.	0	0	0	0	1.503	0
_08630	phage-rel.	0	0	0	0	1.189	0
_08635	phage-rel.	0	0	0	0	1.206	0
_08645	phage-rel.	0	0	0	0	1.083	0
_08660	phage-rel.	0	0	0	0	1.163	0
_08675	phage-rel.	0	0	0	0	1.165	0
_08700	phage-rel.	0	0	0	0	1.125	0
_08715	phage-rel.	0	0	0	0	1.367	0
_12590	phnC	0	0	0	0	-1.166	0
_03890	potD	0	0	0	0	0	1.15
_10780	priA	0	0	0	0	-1.005	0
_06410	prmA	0	0	0	0	3.492	1.62
_05435	pstS	0	0	0	0	-1.246	0
_03750	purD	0	0	0	0	-1.809	-0.981
_03700	purE	0	0	0	0	-0.401	-1.01
_03730	purF	0	0	0	0	-1.469	-1.36
_03745	purH	0	0	0	0	-1.902	-1.16
_03705	purK	0	0	0	0	-0.641	-1.01
_03725	purL	0	0	0	0	-1.269	-1.38
_03735	purM	0	0	0	0	-1.738	-1.12
_03740	purN	0	0	0	0	-1.781	-1.84
_03720	purQ	0	0	0	0	-1.066	-1.21
_03715	purS	0	0	0	0	-0.743	-4.12
_03670	qoxB	0	0	0	0	-1.218	0
_03665	qoxC	0	0	0	0	-1.044	0
_03660	qoxD	0	0	0	0	-1.094	0
_09295	qsrR	0	0	0	0	0.662	-1.68

						Log$_2$ Fold Change	
Locus tag KQU62_	Gene Name	Regulator 1	Reg. 2	Reg. 3	Reg. 4	transcriptome	proteome
_04135	racE	0	0	0	0	1.387	0.408
_06785	radC	0	0	0	0	1.049	0
_04755	rbfA	0	0	0	0	1.027	0.465
_04530	recG	0	0	0	0	0.501	-1.04
_02275	recQ1	0	0	0	0	0.979	-1.54
_05660	rnhA	0	0	0	0	1.665	0
_12305	rnpA	0	0	0	0	1.558	0
_01340	rplA	0	0	0	0	1.628	0
_09940	rplB	0	0	0	0	1.053	0
_09950	rplD	0	0	0	0	1.065	0
_01335	rplK	0	0	0	0	1.638	0.207
_09945	rplW	0	0	0	0	1.129	0
_12310	rpmH	0	0	0	0	1.489	0
_07095	rpsD	0	0	0	0	1.063	0.433
_09960	rpsJ	0	0	0	0	1.48	0
_04500	rsgA	0	0	0	0	0	-1.05
_06405	rsmE	0	0	0	0	1.837	0
_02000	sarX	0	0	0	0	-4.363	0
_12010	sasA	0	0	0	0	-1.756	-1.48
_12550	sasD	0	0	0	0	-3.247	0
_13150	scdA	0	0	0	0	-3.085	0
_10390	sdpC	0	0	0	0	2.201	0.544
_01455	sdrC	0	0	0	0	-1.246	0
_01460	sdrD	0	0	0	0	-1.844	0
_01465	sdrE	0	0	0	0	-1.583	0
_12005	secY2	0	0	0	0	-1.24	0
_07670	sei	0	0	0	0	1.605	0
_02700	sel	0	0	0	0	-1.6	0
_07675	sem	0	0	0	0	1.944	0
_07660	sen	0	0	0	0	1.141	0
_07680	seo	0	0	0	0	3.286	0
_12355	serS	0	0	0	0	-1.354	-0.404
_07665	seu	0	0	0	0	1.395	0
_06065	spcT	0	0	0	0	1.041	0
_10280	spdB	0	0	0	0	1.502	0
_00710	SPIN	0	0	0	0	-1.774	0
_07595	splF	0	0	0	0	1.248	0
_03325	spxA	0	0	0	0	-0.207	1.98
_04215	ssl12	0	0	0	0	1.089	0
_04220	ssl13	0	0	0	0	1.271	0
_04225	ssl14	0	0	0	0	1.131	0
_03610	sspA	0	0	0	0	-1.033	0
_03605	sspB	0	0	0	0	-1.544	0
_08280	sspB2	0	0	0	0	-1.086	0

						Log$_2$ Fold Change	
Locus tag KQU62_	Gene Name	Regulator 1	Reg. 2	Reg. 3	Reg. 4	transcriptome	proteome
_11040	stbD	0	0	0	0	1.835	0
_05550	sucA	0	0	0	0	-1.303	-0.246
_03020	sufA	0	0	0	0	1.04	0
_10625	tetR	0	0	0	0	1.054	0
_06710	tgt	0	0	0	0	1.084	0
_07955	tRNA-Ala	0	0	0	0	1.611	0
_01200	tRNA-Arg	0	0	0	0	1.003	0
_07965	tRNA-Arg	0	0	0	0	1.96	0
_07695	tRNA-Asn	0	0	0	0	1.321	0
_09515	tRNA-Asn	0	0	0	0	2.472	0
_07715	tRNA-Asp	0	0	0	0	1.735	0
_07920	tRNA-Asp	0	0	0	0	1.288	0
_07935	tRNA-Asp	0	0	0	0	1.584	0
_07885	tRNA-Cys	0	0	0	0	1.903	0
_07890	tRNA-Gln	0	0	0	0	2.116	0
_07690	tRNA-Glu	0	0	0	0	3.06	0
_09510	tRNA-Glu	0	0	0	0	1.316	0
_07880	tRNA-Gly	0	0	0	0	1.771	0
_07975	tRNA-Gly	0	0	0	0	1.401	0
_08980	tRNA-Gly	0	0	0	0	1.242	0
_07705	tRNA-His	0	0	0	0	1.333	0
_07895	tRNA-His	0	0	0	0	1.774	0
_07945	tRNA-Ile	0	0	0	0	1.751	0
_08015	tRNA-Ile	0	0	0	0	1.126	0
_07875	tRNA-Leu	0	0	0	0	1.782	0
_07970	tRNA-Leu	0	0	0	0	1.961	0
_07980	tRNA-Leu	0	0	0	0	1.746	0
_08985	tRNA-Leu	0	0	0	0	1.905	0
_07985	tRNA-Lys	0	0	0	0	2.335	0
_09490	tRNA-Lys	0	0	0	0	1.497	0
_07720	tRNA-Met	0	0	0	0	2.581	0
_07925	tRNA-Met	0	0	0	0	2.012	0
_07950	tRNA-Met	0	0	0	0	1.745	0
_07710	tRNA-Phe	0	0	0	0	1.212	0
_07915	tRNA-Phe	0	0	0	0	1.428	0
_07960	tRNA-Pro	0	0	0	0	1.285	0
_07685	tRNA-Ser	0	0	0	0	3.089	0
_07930	tRNA-Ser	0	0	0	0	2.099	0
_07940	tRNA-Ser	0	0	0	0	1.763	0
_01180	tRNA-Thr	0	0	0	0	1.05	0
_07910	tRNA-Thr	0	0	0	0	1.602	0
_07990	tRNA-Thr	0	0	0	0	1.955	0
_07900	tRNA-Trp	0	0	0	0	1.943	0
_07905	tRNA-Tyr	0	0	0	0	1.786	0

Locus tag KQU62_	Gene Name	Regulator 1	Reg. 2	Reg. 3	Reg. 4	Log $_2$ Fold Change transcriptome	proteome
_09500	tRNA-Tyr	0	0	0	0	1.736	0
_01175	tRNA-Val	0	0	0	0	1.894	0
_07995	tRNA-Val	0	0	0	0	2.686	0
_09505	tRNA-Val	0	0	0	0	1.23	0
_09800	truA	0	0	0	0	0.619	-1.9
_02500	trxB	0	0	0	0	1.156	0.29
_06565	udk	0	0	0	0	1.097	0
_07045	uspA1	0	0	0	0	-1.298	0
_01040	veg	0	0	0	0	1.166	0
_02735	vwb	0	0	0	0	1.436	0
_08120	yfkK	0	0	0	0	-1.479	0
_04010	ylbN	0	0	0	0	1.401	0
_11035	yoeB (toxin)	0	0	0	0	1.759	0
_07460	ytkD	0	0	0	0	1.229	0
_01610	ywzC	0	0	0	0	1.5	0
_08810	hld	AgrA	0	0	0	1.049	0
_08835	agrA	AgrA	CodY	0	0	-1.967	0
_03120	argH	ArgR	CodY	0	0	-2.976	0
_07860	artQ	ArgR	CodY	0	0	-3.261	0
_07865	artM	ArgR	CodY	0	0	-3.006	0
_11890	arcR	ArgR	CcpA	Rex	ArcR	-1.403	0
_11895	arcC	ArgR	CcpA	Rex	ArcR	-1.34	0
_11900	arcD	ArgR	CcpA	Rex	ArcR	-2.621	0
_11905	arcB	ArgR	CcpA	Rex	ArcR	-2.38	0
_11910	arcA	ArgR	CcpA	Rex	ArcR	-3.143	0
_12790	argJ	ArgR	0	0	0	-1.074	0
_12795	argC	ArgR	0	0	0	-1.174	0
_12800	argD	ArgR	0	0	0	-1.254	0
_13185	-	BglR	0	0	0	2.482	0
_13190	bglA	BglR	0	0	0	1.866	0
_01520	vraA	BirA	0	0	0	1.529	0
_01530	vraC	BirA	0	0	0	1.101	0
_01535	vraP	BirA	0	0	0	1.74	0
_00215	glpT	CcpA	0	0	0	-1.59	0
_01400	kbl	CcpA	0	0	0	-1.252	0
_07175	acuA	CcpA	CodY	0	0	-1.047	0
_07340	putA	CcpA	0	0	0	-1.837	0
_08245	putP	CcpA	0	0	0	-1.228	0
_10440	gltS	CcpA	0	0	0	1.758	0
_11210	gntP	CcpA	GntR	0	0	-1.066	0
_11215	gntK	CcpA	GntR	0	0	-1.082	0
_11220	gntR	CcpA	GntR	0	0	-2.658	0
_11940	manR	CcpA	0	0	0	-1.023	0

						Log$_2$ Fold Change	
Locus tag KQU62_	Gene Name	Regulator 1	Reg. 2	Reg. 3	Reg. 4	transcriptome	proteome
_12090	lip	CcpA	0	0	0	-2.027	0
_12350	hutH	CcpA	0	0	0	-1.093	0
_12825	ptsG	CcpA	0	0	0	-2.246	-0.335
_12940	uhpT	CcpA	0	0	0	-1.537	0
_12960	pflB	CcpA	Rex	0	0	-4.222	0
_02310	dtpT	CodY	0	0	0	-1.414	-0.277
_02435	raiA	CodY	0	0	0	-1.029	0
_03125	argG	CodY	ArgR	0	0	-2.811	0
_03485	-	CodY	0	0	0	-1.73	0
_05315	trpE	CodY	T-box	0	0	1.333	0
_05675	-	CodY	0	0	0	-4	0
_05680	tdcB	CodY	0	0	0	-4.544	0
_06285	sodA	CodY	0	0	0	1.377	0.659
_08915	ilvD	CodY	0	0	0	-1.262	0
_08920	ilvB	CodY	0	0	0	-1.463	0
_08925	ilvN	CodY	0	0	0	-1.981	0
_08930	ilvC	CodY	0	0	0	-2.007	0
_08935	leuA	CodY	0	0	0	-1.886	0
_08940	leuB	CodY	0	0	0	-1.136	0
_08950	leuD	CodY	0	0	0	-1.202	0
_10970	opuCD	CodY	0	0	0	-1.651	0
_10975	opuCC	CodY	0	0	0	-1.749	0
_10980	opuCB	CodY	0	0	0	-1.926	0
_10985	opuCA	CodY	0	0	0	-1.981	-0.347
_11710	-	CodY	0	0	0	-1.442	0
_11715	gabT	CodY	0	0	0	-1.618	0
_11720	-	CodY	SigB	0	0	-1.843	0
_11925	aur	CodY	0	0	0	-1.618	0
_12545	sodM	CodY	0	0	0	-1.052	0
_12625	cap5A	CodY	SigB	0	0	-1.901	0
_12630	cap5B	CodY	SigB	0	0	-1.463	0
_12635	cap5C	CodY	SigB	0	0	-2.086	0
_12640	cap5D	CodY	SigB	0	0	-2.147	0
_12645	cap5E	CodY	SigB	0	0	-1.981	0
_12650	cap5F	CodY	SigB	0	0	-1.825	0
_12655	cap5G	CodY	SigB	0	0	-1.797	0
_12660	cap8I	CodY	SigB	0	0	-1.57	0
_12665	cap8J	CodY	SigB	0	0	-1.293	0
_01255	ctsR	CtsR	0	0	0	2.93	4.18
_01260	mcsA	CtsR	0	0	0	2.887	3.29
_01265	mcsB	CtsR	0	0	0	3.19	3.3
_01270	clpC	CtsR	0	0	0	3.247	2.44
_02520	clpP	CtsR	0	0	0	3.162	1.97
_03195	clpB	CtsR	0	0	0	4.013	4.06

						Log$_2$ Fold Change	
**Locus tag KQU62_**	**Gene Name**	**Regulator 1**	**Reg. 2**	**Reg. 3**	**Reg. 4**	**transcriptome**	**proteome**
_06415	dnaJ	CtsR	HrcA	0	0	3.49	2
_06420	dnaK	CtsR	HrcA	0	0	3.507	1.87
_06425	grpE	CtsR	HrcA	0	0	3.333	1.58
_06430	hrcA	CtsR	HrcA	0	0	3.122	2.27
_08780	groL	CtsR	HrcA	0	0	3.055	1.74
_08785	groES	CtsR	HrcA	0	0	3.169	1.43
_00835	mccA	CymR	0	0	0	1.174	0
_00845	metN1	CymR	0	0	0	-1.86	0
_00850	metP2	CymR	0	0	0	-1.812	0
_00855	gmpC	CymR	0	0	0	-1.78	-0.703
_08865	-	CymR	0	0	0	0	-2.03
_10795	tcyB	CymR	0	0	0	-1.032	0
_10800	tcyA	CymR	0	0	0	-1.059	0
_12750	-	CymR	0	0	0	0	-1.29
_05915	ribU	FMN-box	0	0	0	1.376	0
_07345	ribH	FMN-box	0	0	0	0	-2
_07350	ribA	FMN-box	0	0	0	-1.774	0
_07355	ribB	FMN-box	0	0	0	-1.932	0
_02160	fruR	FruR	CcpA	0	0	-1.315	0
_02165	pfkB	FruR	CcpA	0	0	-1.249	0
_02170	fruA	FruR	CcpA	0	0	-1.07	0
_00260	tatC	Fur	0	0	0	1.509	0
_00265	tatA	Fur	0	0	0	1.021	0
_01895	fhuC	Fur	0	0	0	1.259	0
_04030	isdC	Fur	0	0	0	1.363	0
_04035	isdD	Fur	0	0	0	1.399	0
_04040	isdE	Fur	0	0	0	1.089	0
_04045	isdF	Fur	0	0	0	1.039	0
_09590	htsB	Fur	0	0	0	1.106	0
_09595	htsA	Fur	0	0	0	1.576	0
_12460	sbnA	Fur	0	0	0	0	-1.01
_09440	glmS	glms leader	0	0	0	-2.295	0
_08855	nrgA	GlnR	CodY	0	0	1.74	0
_00890	gltC	GltC	CodY	0	0	1.998	0
_03620	-	GraR	0	0	0	1.439	0
_03675	qoxA	GraR	0	0	0	-1.188	0
_05275	mprF	GraR	0	0	0	-0.285	-1.56
_08795	sdrH	GraR	0	0	0	1.173	0
_12780	-	GraR	0	0	0	-2.054	0
_03035	nhaC	HisR	0	0	0	1.815	0
_10525	hrtB	HssR	0	0	0	1.205	0
_01345	rplJ	L leader	0	0	0	-1.097	0
_04600	rplS	L leader	0	0	0	1.734	0.344
_09690	lacA	LacR	0	0	0	0	2.46

						Log$_2$ Fold Change	
**Locus tag KQU62_**	**Gene Name**	**Regulator 1**	**Reg. 2**	**Reg. 3**	**Reg. 4**	**transcriptome**	**proteome**
_12900	malK	MalR	CcpA	0	0	-2.113	0
_00200	mepR	MepR	0	0	0	1.443	0
_00205	mepA	MepR	0	0	0	2.955	0
_01825	mntA	MntR	0	0	0	0	-1.27
_03905	mntH	MntR	0	0	0	-0.667	1.52
_09445	mtlF	MtlR	0	0	0	-1.129	0
_12830	mupG	MurR	CcpA	0	0	-1.421	0
_12835	murQ	MurR	0	0	0	-1.162	0
_00105	nanA	NanR	CcpA	0	0	-1.09	0
_11790	nrdG	NrdR	0	0	0	-1.231	0
_11795	nrdD	NrdR	0	0	0	-1.14	0
_01240	pdxS	PdxR	0	0	0	-1.07	-0.395
_01245	pdxT	PdxR	0	0	0	-1.27	-0.431
_02230	queC	PreQ1 riboswitch	0	0	0	1.656	0
_05655	-	PreQ1 riboswitch	0	0	0	1.037	0
_04390	uraA	pyrR leader	0	0	0	-2.236	0
_04395	pyrB	pyrR leader	0	0	0	-2.825	0
_04400	pyrC	pyrR leader	0	0	0	-2.233	-0.504
_04405	carA	pyrR leader	0	0	0	-2.402	-0.639
_04410	carB	pyrR leader	0	0	0	-2.615	-0.601
_04415	pyrF	pyrR leader	0	0	0	-2.882	0
_04420	pyrE	pyrR leader	0	0	0	-2.61	0
_04425	-	pyrR leader	0	0	0	-2.562	0
_00055	lldP	Rex	0	0	0	-2.286	0
_01680	adhP	Rex	0	0	0	-2.009	0
_05685	ald	Rex	0	0	0	-1.414	0
_10570	lctP_2	Rex	0	0	0	-2.477	0
_10680	narK	Rex	NreC	0	0	-6.735	0
_10695	nreC	Rex	NreC	0	0	-1.326	-0.565
_10700	nreB	Rex	NreC	0	0	-1.743	-0.524
_10705	nreA	Rex	NreC	0	0	-2.249	0
_10710	narI	Rex	NreC	0	0	-5.24	-1.24
_10715	narJ	Rex	NreC	0	0	-6.649	0
_10720	narH	Rex	NreC	0	0	-7.31	0
_10725	narG	Rex	NreC	0	0	-5.854	0
_10730	nasF	Rex	NreC	0	0	-7.124	0
_10735	nasE	Rex	NreC	0	0	-7.628	0
_10740	nasD	Rex	NreC	0	0	-7.174	0
_10745	nirR	Rex	NreC	0	0	-6.783	0
_11145	-	Rex	0	0	0	-3.032	0
_11320	ddh	Rex	0	0	0	-1.735	0
_12620	adhE	Rex	0	0	0	-3.104	0

						Log$_2$ Fold Change	
Locus tag KQU62_	Gene Name	Regulator 1	Reg. 2	Reg. 3	Reg. 4	transcriptome	proteome
_12965	pflA	Rex	CcpA	0	0	-4.824	0
_13055	ldh1/ lctE	Rex	0	0	0	-2.9	0
_07325	rot	RNAIII	0	0	0	-1.505	0
_08470	sak	SaeR	0	0	0	-1.048	0
_08745	lukF	SaeR	0	0	0	-1.183	0
_12935	-	SaeR	0	0	0	-1.653	0
_02855	metN	S-box	0	0	0	-1.232	-0.78
_02860	metI2	S-box	0	0	0	-1.281	0
_02865	metQ	S-box	0	0	0	-1.557	-0.614
_01760	-	SigB	0	0	0	2.678	0
_01765	mnhA2	SigB	0	0	0	1.743	0
_01770	mnhB2	SigB	0	0	0	1.694	0
_01775	mnhC2	SigB	0	0	0	1.805	0
_01780	mnhD2	SigB	0	0	0	1.765	0
_01785	mnhE	SigB	0	0	0	1.573	0
_01790	mnhF2	SigB	0	0	0	1.708	0
_01795	mnhG2	SigB	0	0	0	1.602	0
_02220	queE	SigB	PreQ1 riboswitch	0	0	1.504	0
_02225	queD	SigB	PreQ1 riboswitch	0	0	1.567	0
_02515	whiA	SigB	0	0	0	0.609	1.32
_02915	mpfB	SigB	0	0	0	1.393	0
_02920	fabK	SigB	0	0	0	1.506	0
_03560	-	SigB	0	0	0	1.378	0
_07025	-	SigB	0	0	0	-1.004	-0.335
_08095	-	SigB	0	0	0	-0.223	1.19
_08755	-	SigB	0	0	0	-1.06	0
_09380	-	SigB	0	0	0	-1.095	0
_09400	-	SigB	0	0	0	-1.024	0
_09460	mtlD	SigB	MtlR	0	0	-1.359	0
_09630	sspC	SigB	0	0	0	-1.141	0
_09635	amaP	SigB	0	0	0	-1.008	0
_09640	opuD2	SigB	0	0	0	-1.496	0
_09755	map-w	SigB	0	0	0	-1.022	0
_10580	-	SigB	0	0	0	-1.002	-0.542
_11445	clpL	SigB	0	0	0	-1.527	0
_11515	crtN	SigB	0	0	0	-0.64	-1.25
_11530	crtI	SigB	0	0	0	-1.016	0
_11535	crtO	SigB	0	0	0	-1.13	0
_00910	treP	TreR	CcpA	0	0	-1.116	0
_00915	treC	TreR	CcpA	0	0	-0.53	-1.36
_00860	sle1	WalR	0	0	0	1.756	0.368
_01985	amiD7	WalR	0	0	0	1.133	0

						Log$_2$ Fold Change	
**Locus tag KQU62_**	**Gene Name**	**Regulator 1**	**Reg. 2**	**Reg. 3**	**Reg. 4**	**transcriptome**	**proteome**
_03635	atl	WalR	SigB	0	0	2.279	0
_09160	sceD	WalR	0	0	0	2.22	0
_10205	ssaA	WalR	GraR	0	0	2.439	0
_10230	ssaA2	WalR	0	0	0	3.989	0
_11425	amiD2	WalR	0	0	0	1.768	0
_11540	ssaA1	WalR	RNAIII	0	0	1.558	0
_11550	isaA	WalR	0	0	0	1.329	0
_13245	lytM	WalR	0	0	0	2.259	0

Table S4: Strains and plasmids used in this study.

Strain	Description/ Genotype	Source
Bacillus subtilis		
B. subtilis 168	*trpC2*	5, 6
B. subtilis 168 ΔclpP (QB4916)	Derivative of *B. subtilis 168*; deletion of *clpP*; spectinomycin resistant	7
Escherichia coli		
E. coli BL21 LOBSTR	Derived from *E. coli* BL21(DE3); modified copies of *arnA* and *slyD*	8
E. coli K12 JM109	*recA*1, *endA*l, *gyrA*96, *thi*, *hsdR*17, *supE*44, *relA*1, λ^-, Δ(*lac-proAB*), [F', *traD*36, *proAB*, iacI^{q}ZΔM15]	9
Staphylococcus aureus		
HG003	Derivative of NCTC 8325; *rsbU*$^+$, *tcaR*$^+$	10
NCTC8325-4	Laboratory strain; derivative of NCTC 8325; *rsbU*$^-$, *agr*$^-$, UV-cured of Φ11, Φ12, Φ13 prophages	11
NCTC8325-4 ΔrecU	Derivative of *S. aureus* NCTC8325-4; *recU* mutant lacking initial 165 codons of the gene	12
RN4220	Laboratory strain; derivative of NCTC 8325-4; *rsbU*$^-$, *agr*$^-$, UV-cured of Φ11, Φ12, Φ13 prophages; restriction-negative	13
RN4220 GyrA^{S84L}	Derivative of *S. aureus* RN4220; ciprofloxacin-resistant (GyrA Ser84Leu)	14
RN4220 GyrB^{G86S}	Derivative of *S. aureus* RN4220; novobiocin-resistant (GyrB Gly86Ser)	14
SG511 Berlin	Antibiotic-susceptible strain (CP076660, locus tag prefix KQU62_)	RKI Berlin, Germany
SA113	Derivative of NCTC 8325; restriction- and modification-deficient	15
SA113 ΔtagO	Derivative of *S. aureus* SA113; erythromycin-resistant	16
**Plasmid**	**Description**	**Source**
pEPSA5	Down-regulation of essential *S. aureus* genes; host *S. aureus* RN4220 by antisense RNA; clones containing inserts targeting *gyrA*, *gyrB*, *parC*, *parE*, *topA*, *topB*, and *divIVA*	17
pET19-gyrA	Plasmid for over-expression of full-length GyrA endonuclease from *S. aureus* SG511	this study
pET21-gyrB	Plasmid for over-expression of full-length GyrB endonuclease from *S. aureus* SG511	this study
pREP4groESL(MT)	Plasmid encoding *E. coli* JM109 GroESL chaperones; facilitates folding and avoids formation of inclusion bodies during protein over-expression	18
pTX15	Xylose-inducible vector, containing *xylA* and *xylR* for expression control, TetR	19
pTX15-vicRK	Derivative of pTX15 with *walRK* under control of XylR	20

Table S5: Oligonucleotide primers used in this study; n.d., not determined.

Primer	Sequence 5‘-3‘and restriction sites	T_A [°C]	Reference
gyrA-for	CTATACTCTAACTCGAGGCTGAATTACCTCAATC (XhoI)	56.8	21
gyrA-rev	CATTACACATCCTCGAGTTATTATTCTTCATCTG (XhoI)	56.8	21
gyrB-for	CGCGGATCCAATTTTGTTTAACTTTAAGAAGGAG ATATAGCATGGTGACTGCATTGTCA (BamHI)	65.0	21
gyrB-rev	TGCCTCGAGAGAACCCATGGTGAAGTCTAAGTTT GCATAAACTGC (XhoI)	65.0	21
R1-for (V-448594)	TGAGGCAAACAAAACAACTCA	62.0	22
R1-rev	AGCCATCTTTCTTTGTGTTAACC	62.0	This study
R2-for (V-92795f)	AATTGAATAACGGGAAGTAGCTCAG	62.0	22
R2-rev	GTTTCGTCAGATTCAAACGTTTTC	62.0	This study
R3-for	GAATAAATGGTGGAGAATGAC	57.5	This study
R3-rev	CGCGTTATAATTAAATCTTGTAAG	57.5	This study
R4-for (V-98307r)	GCTTGCTTCTTTCCTCTCCTTC	62.0	22
R4-rev	ACCTCAGCCTTCCAAGCTGATG	62.0	This study
R5-for	CGTGTTACCGCCGTGAAAGG	58.4	This study
R5-rev_2	TTATTGTTATTGACTTCAATAATTAAATGAAGTAC	58.4	This study
V-450333	AGGTAGCCGTATCGGAGGT	n.d.	22
V-451191	GGAGGACCATCTCCTAAGGCTA	n.d.	22
V-452112f	GGGTTAGTCGGGTCCTAAGC	n.d.	22

Publication bibliography

1. Schneider CA, Rasband WS, Eliceiri KW. 2012. NIH Image to ImageJ: 25 years of image analysis. Nature Meth 9:671–675. doi:10.1038/nmeth.2089.
2. Jones MB, Nierman WC, Shan Y, Frank BC, Spoering A, Ling L, Peoples A, Zullo A, Lewis K, Nelson KE. 2017. Reducing the bottleneck in discovery of novel antibiotics. Microb Ecol 73:658–667. doi:10.1007/s00248-016-0889-3.
3. Wickham H. 2016. ggplot2. Elegant Graphics for Data Analysis. Springer.
4. Fuchs S, Mehlan H, Bernhardt J, Hennig A, Michalik S, Surmann K, Pané-Farré J, Giese A, Weiss S, Backert L, Herbig A, Nieselt K, Hecker M, Völker U, Mäder U. 2018. AureoWiki‑the repository of the *Staphylococcus aureus* research and annotation community. Int J Med Microbiol 308:558–568. doi:10.1016/j.ijmm.2017.11.011.
5. Burkholder PR, Giles NH. 1947. Induced biochemical mutations in *Bacillus subtilis*. Am J Bot 34:345. doi:10.2307/2437147.
6. Spizizen J. 1958. Transformation of biochemically deficient strains of *Bacillus subtilis* by deoxyribonucleate. PNAS 44:1072–1078. doi:10.1073/pnas.44.10.1072.
7. Msadek T, Dartois V, Kunst F, Herbaud ML, Denizot F, Rapoport G. 1998. ClpP of *Bacillus subtilis* is required for competence development, motility, degradative enzyme synthesis, growth at high temperature and sporulation. Mol Microbiol 27:899–914. doi:10.1046/j.1365-2958.1998.00735.x.
8. Andersen KR, Leksa NC, Schwartz TU. 2013. Optimized *E. coli* expression strain LOBSTR eliminates common contaminants from His-tag purification. Proteins 81:1857–1861. doi:10.1002/prot.24364.
9. Yanisch-Perron C, Vieira J, Messing J. 1985. Improved M13 phage cloning vectors and host strains: nucleotide sequences of the M13mp18 and pUC19 vectors. Gene 33:103–119. doi:10.1016/0378-1119(85)90120-9.
10. Herbert S, Ziebandt A-K, Ohlsen K, Schäfer T, Hecker M, Albrecht D, Novick R, Götz F. 2010. Repair of global regulators in *Staphylococcus aureus* 8325 and comparative analysis with other clinical isolates. Inf Immun 78:2877–2889. doi:10.1128/IAI.00088-10.
11. Novick R. 1967. Properties of a cryptic high-frequency transducing phage in *Staphylococcus aureus*. Virology 33:155–166. doi:10.1016/0042-6822(67)90105-5.
12. Pereira AR, Reed P, Veiga H, Pinho MG. 2013. The Holliday junction resolvase RecU is required for chromosome segregation and DNA damage repair in *Staphylococcus aureus*. BMC Microbiol 13:18. doi:10.1186/1471-2180-13-18.
13. Kreiswirth BN, Löfdahl S, Betley MJ, O'Reilly M, Schlievert PM, Bergdoll MS, Novick RP. 1983. The toxic shock syndrome exotoxin structural gene is not detectably transmitted by a prophage. Nature 305:709–712. doi:10.1038/305709a0.
14. Chiriac AI, Kloss F, Krämer J, Vuong C, Hertweck C, Sahl H-G. 2015. Mode of action of closthioamide. J Antimicrob Chemother 70:2576–2588. doi:10.1093/jac/dkv161.
15. Iordanescu S, Surdeanu M. 1976. Two restriction and modification systems in *Staphylococcus aureus* NCTC8325. J Gen Microbiol 96:277–281. doi:10.1099/00221287-96-2-277.
16. Weidenmaier C, Kokai-Kun JF, Kristian SA, Chanturiya T, Kalbacher H, Gross M, Nicholson G, Neumeister B, Mond JJ, Peschel A. 2004. Role of teichoic acids in *Staphylococcus aureus* nasal colonization, a major risk factor in nosocomial infections. Nat Med 10:243–245. doi:10.1038/nm991.
17. Forsyth RA, Haselbeck RJ, Ohlsen KL, Yamamoto RT, Xu H, Trawick JD, Wall D, Wang L, Brown-Driver V, Froelich JM, C KG, King P, McCarthy M, Malone C, Misiner B, Robbins D, Tan Z, Zhu Zy

Z, Carr G, Mosca DA, Zamudio C, Foulkes JG, Zyskind JW. 2002. A genome-wide strategy for the identification of essential genes in *Staphylococcus aureus*. Mol Microbiol 43:1387–1400. doi:10.1046/j.1365-2958.2002.02832.x.

18. Türck M, Bierbaum G. 2012. Purification and activity testing of the full-length YycFGHI proteins of *Staphylococcus aureus*. PloS One 7:e30403. doi:10.1371/journal.pone.0030403.
19. Peschel A, Ottenwälder B, Götz F. 1996. Inducible production and cellular location of the epidermin biosynthetic enzyme EpiB using an improved staphylococcal expression system. FEMS Microbiol Lett 137:279–284. doi:10.1111/j.1574-6968.1996.tb08119.x.
20. Jansen A, Türck M, Szekat C, Nagel M, Clever I, Bierbaum G. 2007. Role of insertion elements and *yycFG* in the development of decreased susceptibility to vancomycin in *Staphylococcus aureus*. Int J Med Microbiol 297:205–215. doi:10.1016/j.ijmm.2007.02.002.
21. Saiki AY, Shen LL, Chen CM, Baranowski J, Lerner CG. 1999. DNA cleavage activities of *Staphylococcus aureus* gyrase and topoisomerase IV stimulated by quinolones and 2-pyridones. Antimicrob Agents Chemother 43:1574–1577. doi:10.1128/AAC.43.7.1574.
22. Berscheid A. 2014. Characterization of *Staphylococcus aureus* VC40 reveals a major role of the VraS and WalK histidine kinases in the development of vancomycin and daptomycin resistance. PhD thesis, Rheinische Friedrich-Wilhelms-Universität, Bonn, Germany.
